# Supplementary material for: From the floret to the canopy: High temperature tolerance during flowering
Source: Plant Commun. 2023 May 23;4(6):100629. doi: 10.1016/j.xplc.2023.100629 (PMC10721465; doi:10.1016/j.xplc.2023.100629)
Supplement: Document S1 — . Figures S1–S3 and Tables S1–S3 [file mmc1.pdf]

Plant Communications, Volume *4*

Supplemental information

From the floret to the canopy: High temperature tolerance during flowering

Mayang Liu, Yuhan Zhou, Jiaxin Sun, Fen Mao, Qian Yao, Baole Li, Yuanyuan Wang, Yingbo Gao, Xin Dong, Shuhua Liao, Pu Wang, and Shoubing Huang

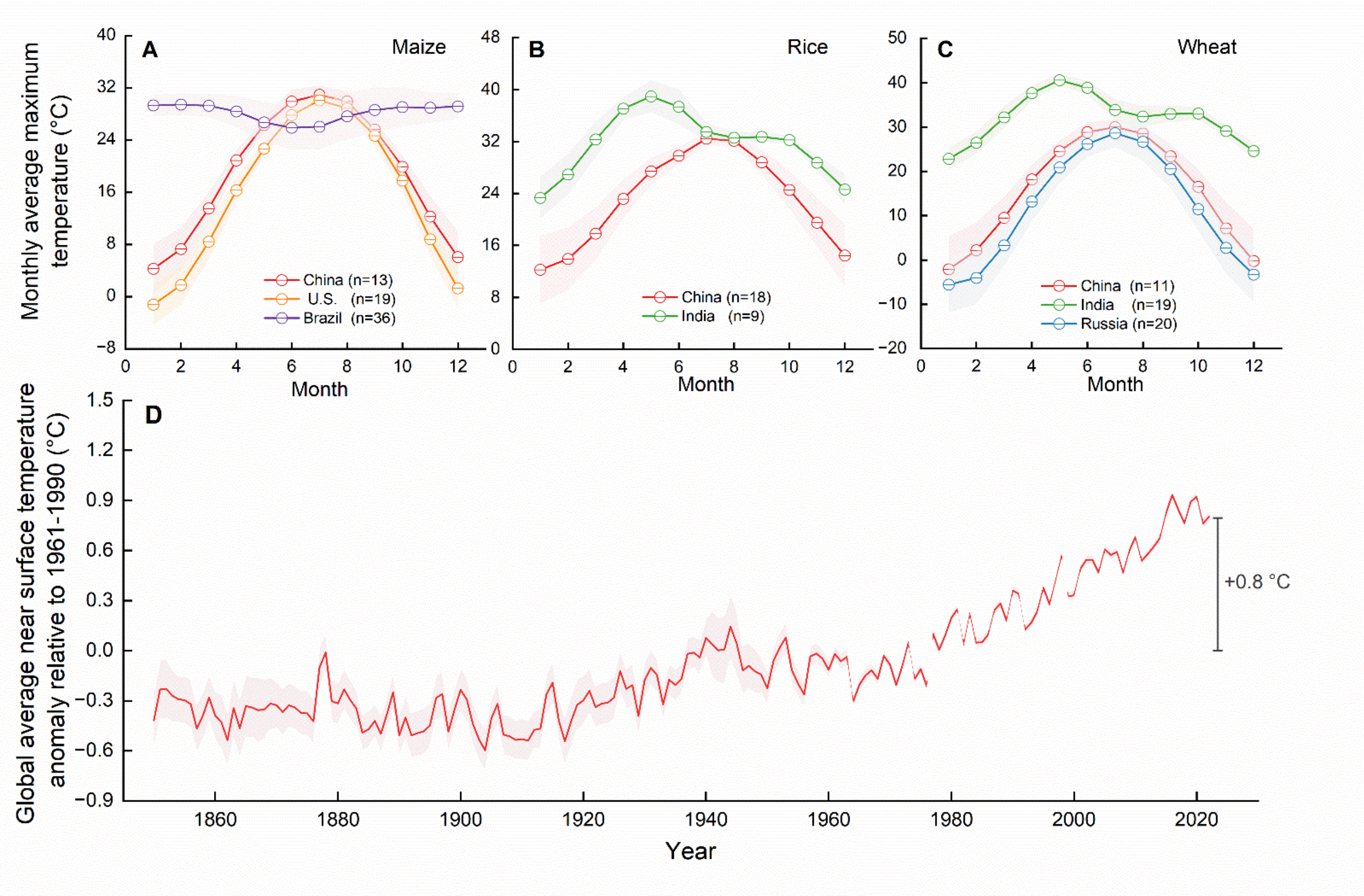

Supplementary Figure S1. Maximum temperature varies on monthly level and global average near surface temperature rises year-by-year. (A-C) Monthly average maximum temperature fluctuation in the major production countries of maize (China, U. S. and Brazil), rice (China and India) and wheat (China, India and Russia). Data were plotted from the mean of monthly recorded maximum temperature from 1960 to 2018 in local meteorological stations (the "n" represents the number of stations selected in each country). Data were retrieved from https://www.worldclim.org/data/monthlywth. The shaded areas indicated the margin of standard deviation (SD) in averaging the monthly mean maximum temperatures recorded at several stations in each country. (D) Global average near surface temperature anomaly relative to 1961-1990. The HadCRUT5 near surface temperature dataset provided by Met Office Hadley Centre and Climatic Research Unit (Morice et al., 2012), and retrieved from https://hadleyserver.metoffice.gov.uk/hadcrut5. Recorded interval of 1850-2022. The light pink area and lower limit and upper limit denote the 95% confidence interval of the HadCRUT5 dataset.

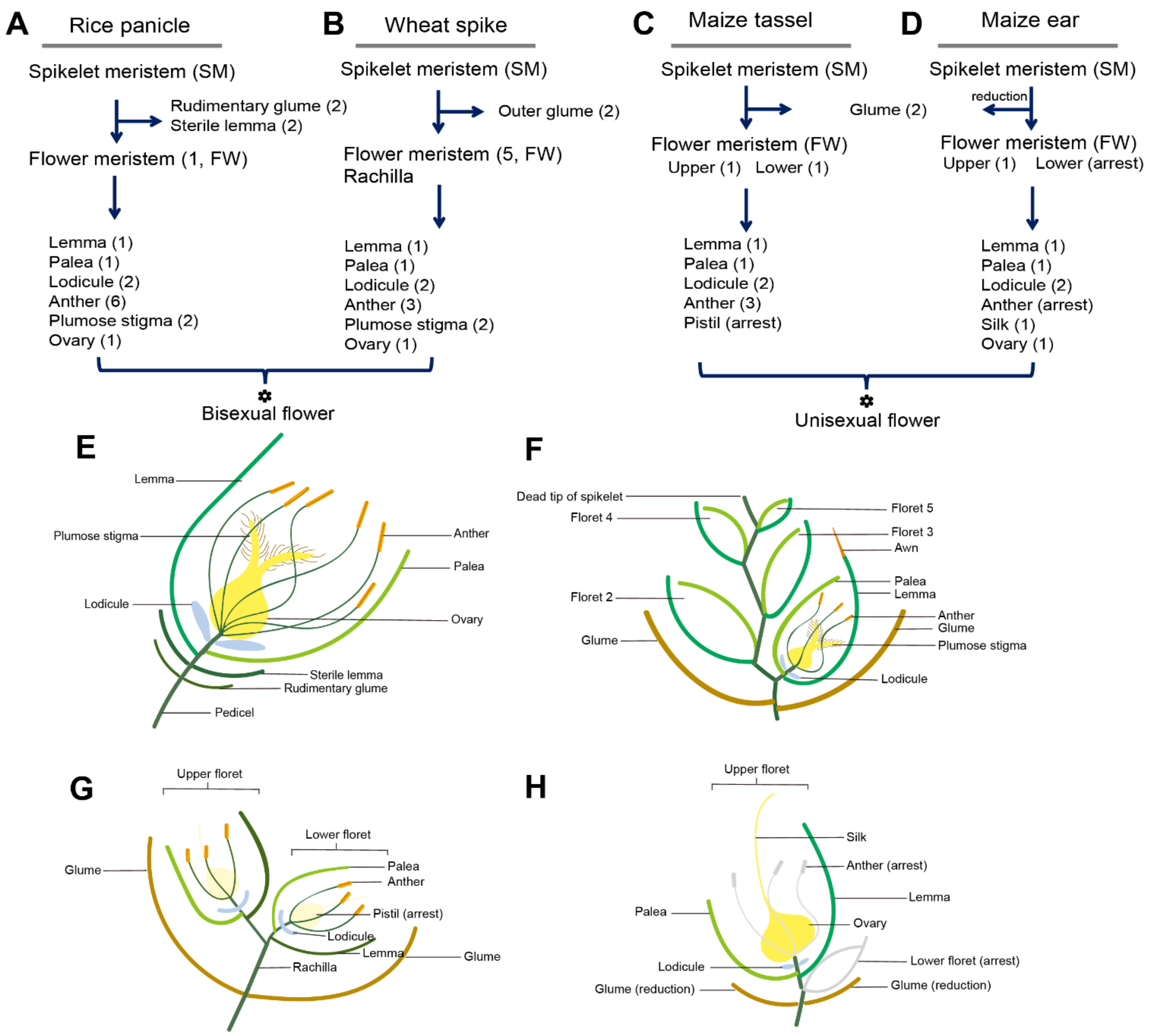

Supplementary Figure S2. Spikelet (or floret) development in rice, wheat and maize under normal growth environment. (A – D) Meristem transition and lateral organ differentiation of spikelet on the rice panicle, wheat spike, maize tassel and ear, which are summarized based on Riechmann & Wellmer (2014), with modifications. (E – H) Schematic representation of the bisexual floret in rice (E) and wheat (F), and the unisexual floret in maize tassel (G) and ear (H), which are drawn based on McSteen, Laudencia-Chingcuanco, & Colasanti (2000), Thompson & Hake (2009), Morojele & Burger (2009), with modifications. The florets are composed of the glume, lemma, palea, lodicules, and male and female reproduction organ (i.e., stamens and pistil). During sexual differentiation, some of the floret tissues undergo different degrees of degeneration or even abortion, e.g., rudimentary glume, and sterile lemma of another abortion floret in rice; arrested pistil of unisexual floret in maize tassel; reduced glume, arrested lower floret, and arrested anther and style of upper floret in maize ear.

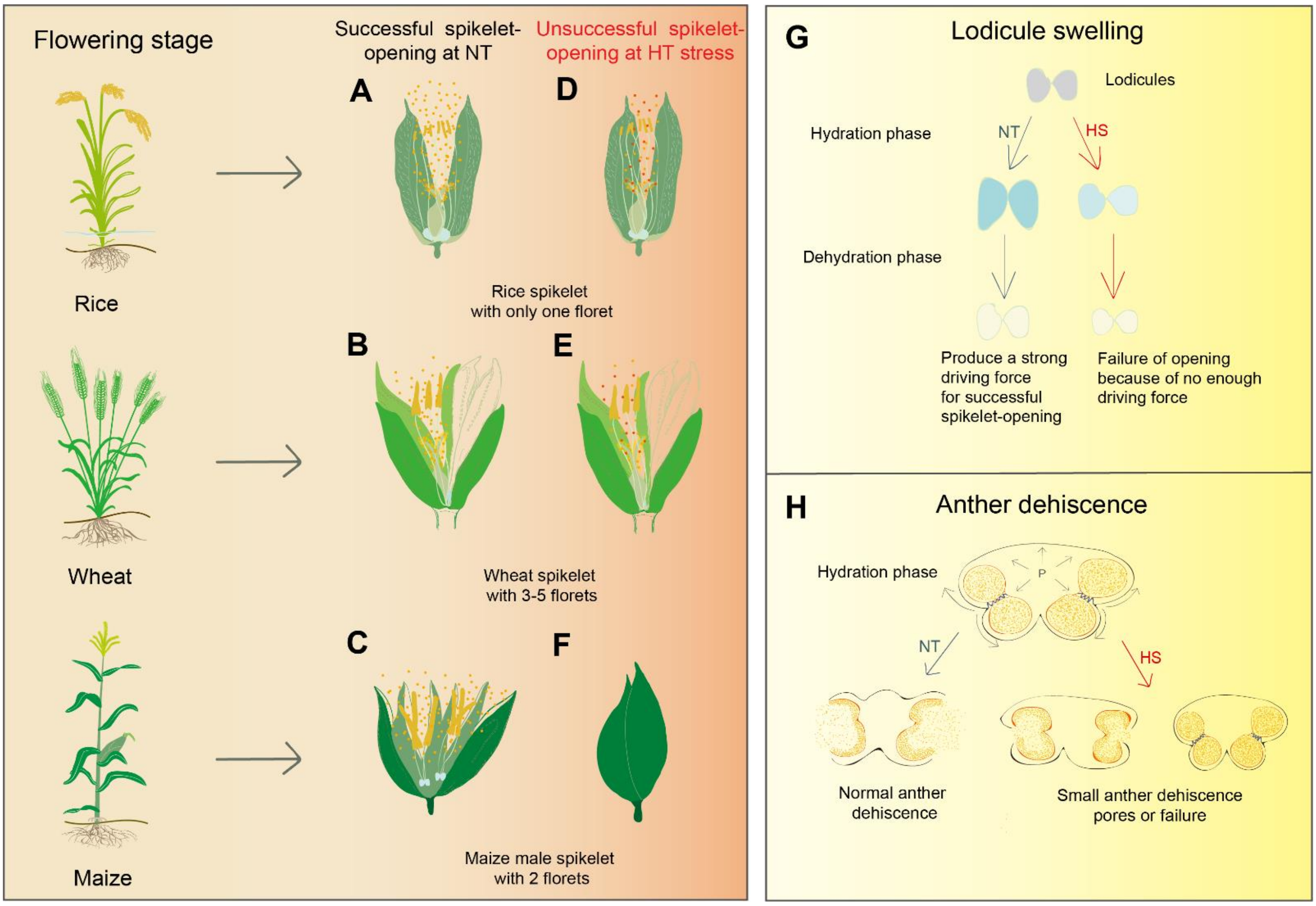

Supplementary Figure S3. Moderate or extreme high temperature (HT) stress affects spikelet-opening and pollen-shedding of rice, wheat and maize at flowering stage. (A-F) illustrate the morphological differences of the spikelet-opening at normal temperature (NT) and HT stress, respectively. HT stress results in unsuccessful spikelet-opening, including smaller opening angle or even opening failure, defective anther dehiscence, non-viable pollen grains (viable: pale yellow; nonviable: red) and reduced pollen shedding number. (G) depicts the distinct hydration and dehydration states of spikelet (floret) lodicules under NT and HT stress. During the spikelet-opening, the lodicules absorb enough water and swell resulting in a strong driving force to sperate lemma and palea of spikelet, which is significantly limited under HT stress. (H) clearly illustrates the pressure characteristic of anther at hydration stage (the driving force for dehiscence) and the various extents of anther dehiscence under NT and HT stress. HT stress induces the defective anther dehiscence with small dehiscence pore or even failure. This graphic was drawn based on Beauzamy, Nakayama, & Boudaoud (2014), with modifications.

Supplementary Table S1. Parameters of nonlinear Boltzmann model for fitting seed set ratio (%) of rice, wheat, and maize in response to high daytime and nighttime temperatures.

**Parameter**	**SS_HDT**			**SS_HNT**		
	Rice	Wheat	Maize	Rice	Wheat	Maize
SS_A_1	81.3(1.5)	82.4(1.7)	80.3(1.4)	81.9(1.1)	82.1(2.0)	84.6(1.2)
SS_A_2	32.0(2.9)	57.0(1.8)	37.9(3.6)	31.1(3.0)	62.6(5.2)	64.4(2.3)
$TT_{cri}\_X_0$	37.2(0.2)	27.3(0.5)	37.9(0.4)	31.2(0.2)	19.6(2.7)	27.3(1.6)
R^2	0.55	0.42	0.60	0.66	0.32	0.47
p_value	*<0.000*	*<0.000*	*<0.000*	*<0.000*	*<0.000*	*<0.000*
n	386	175	202	205	56	76

Note: Values in the bracket are the standard error. The critical temperature threshold is the temperature level during flowering that can significantly change the seed set ratio. Boltzmann model is as follows:

$$y = A_2 + (A_1 - A_2)/(1 + \exp((x - x_0)/dx)$$

where A_1 and A_2 are the initial and final values (i.e., SS ratio) of the fit curve, X is the dependent variable (i.e., temperature level), and X_0 is the threshold coefficient (i.e., TT_{cri}).

Supplementary Table S2. Seed set ratio (%) of rice, wheat, and maize when temperature exceeds high daytime and nighttime temperature thresholds (HDT and HNT) in different duration (i.e., hour, day, and week).

Species	Temperature threshold	Max. temperature	Duration of treatment	Seed set (%)	Number of data
**Rice**	HDT>37.2°C	37.5°C	6d	66.3% (44.1~85.3%)	9
		38°C	4-6h	53.5% (25.2~82.6%)	16
			2-6d	46.5% (3.8~85.7%)	48
		39°C	2-4d	15.9% (0.6~71.5%)	29
			2w	58.7% (31.5~84.0%)	12
		40°C	5-6d	35.8% (13.2~62.1%)	15
			1-2w	24.3% (10.0~40.2%)	24
	HNT>31.2°C	32°C	3d	66.6% (66.5~66.6%)	2
			>2w	42.7% (11.8-86.1%)	20
		35°C	>2w	31.5% (10.9-59.7%)	20
**Wheat**	HDT>27.3°C	30°C	5d	90.3% (86.4~99.0%)	5
		35°C	6-7d	77.9% (62.2~89.7%)	3
		31~35°C	>1w	58.5% (20.2~79.6%)	58
	HNT>19.6°C	20~24°C	<1w	72.4% (64.9~89.3%)	5
		20~23°C	>1w	65.0% (60.4~69.7%)	2
**Maize**	HDT>37.9°C	38~39°C	>2w	48.0% (7.6~81.5%)	62
		40~42°C	5d	67.0% (56.6~75.3%)	6
			>2w	29.3% (3.0~71.1%)	21
	HNT>27.3°C	30°C	2w	64.5% (58.2~74.0%)	10

Note: seed set ratio (%) is the mean value of multiple data across the above conditions. Values in bracket are the range of the mean

Supplementary Table S3. Flowering trait, grain yield, and fertility of spikelet and/or floret of rice, wheat and maize under different temperature levels around flowering. Data are derived from temperature-controlled experiments.

Species	Trait	Experimental conditions	High temperature region (Day/night temperature)	Stage of treatment	Duration of temperature	Grain yield (GY)/spikelet/floret fertility (SF/FF) loss relative to normal	Reference
Rice	Spikelet-opening	phytotron	39°C for 2h/26°C	*Ca.* 50% of plans at heading	48h	21%~53.4% of GY	Chen et al., 2020;
	Flowering Peak	Growth cabinet	35°C, 38°C for 11h	Anthesis	1d	7% per °C > 29.6°C or 2.4% per °C >33°C of SF	Jagadish et al., 2007
	Anther dehiscence	Sun-lit phytotron	37.5°C for 6h/26°C	Middle heading	6d	9.3%~49.3% of SF	Matsui et al., 2002
		Growth chamber	37°C, 39°C for 7h	After flowering	3d	/	Matsui et al., 2019
	Stigma elongation	Temperature-controlling greenhouse	35-36°C for 3h/24-27°C	Before anthesis	7d	16.1%~ 38.4% of SF at whole panicles	Wu et al.,2019;
	Pollen production	Temperature-gradient greenhouse	Ambient+5°C	Sowing	/	9.3%~77.7% of SF	Prasad et al., 2006
	Pollination	Sun-lit phytotron	40°C for 6h/26°C	Middle heading	6d	43.1%~79.7% of SF	Matsui et al., 2001

**Wheat**	Time of flowering	Growth chamber	34°C for 7h/16°C	Start of flowering	10d	22%~38% of GY	Aiqing et al., 2018
	Floret morphology	Growth chamber	36°C for 8h/26°C	Onset of booting	5d	FF decreased from 85% at 24°C to 0% at 35°C	Prasad et al., 2014
	Male morphology	Controlled environment cabinet	30°C/30°C, 30°C/20°C	Onset of meiosis in the pollen mother cells	3d	25% of grain set at 30°C/20°C; 37.4% at 30°C/30°C	Saini et al., 1982, 1984,
	Female morphology and fertility	Controlled environment cabinet	30°C/30°C	Onset of meiosis in the pollen mother cells	3d	/	Saini et al., 1983
**Maize**	Flowering dynamics	Environment-controlled greenhouse	40°C for 2h/30°C	V14 stage	14d	73.6% of GY	Wang et al., 2019
		Sun-lit phytotron	40°C at ear level for 4h	15 days before anthesis	~15d	51.8% of kernels loss	Edreira et al., 2011
	Pollen development	Growth chamber	35°C/25°C	Tetrad stage of pollen development	2d	A strong reduction in the number of seeds per cob	Begcy et al., 2019
	Pollen germination						
	Pollen-stigma interaction	Incubator	38°C/22°C	The application of pollen to the silks	2d	15%~18.6% of swelling kernels and 14.1%~17% of formed kernels	Mitchell et al., 1988

	Pollination	Environmental chamber	40°C	/	2 to 24 h after pollination	No fertilization occurs after 6 h of stress	Dupuis et al., 1990
