# Supplementary material for: From the floret to the canopy: High temperature tolerance during flowering
Source: Plant Commun. 2023 May 23;4(6):100629. doi: 10.1016/j.xplc.2023.100629 (PMC10721465; doi:10.1016/j.xplc.2023.100629)
Supplement: Document S2 — . Article plus supplemental information [file mmc2.pdf]

Plant Communications
Review article

CellPress Partner Journal

From the floret to the canopy: High temperature tolerance during flowering

Mayang Liu[1], Yuhan Zhou[1], Jiaxin Sun[1], Fen Mao[1], Qian Yao[1], Baole Li[1], Yuanyuan Wang[2], Yingbo Gao[3], Xin Dong[4], Shuhua Liao[1], Pu Wang[1] and Shoubing Huang[1,*]

[1]College of Agronomy and Biotechnology, China Agricultural University, Beijing, China

[2]College of Agronomy, South China Agricultural University, Guangdong, China

[3]Shandong Academy of Agricultural Sciences, Jinan, China

[4]Chongqing Academy of Agricultural Sciences, Chongqing, China

***Correspondence: Shoubing Huang (huangshoubing@cau.edu.cn)**

https://doi.org/10.1016/j.xplc.2023.100629

ABSTRACT

Heat waves induced by climate warming have become common in food-producing regions worldwide, frequently coinciding with high temperature (HT)-sensitive stages of many crops and thus threatening global food security. Understanding the HT sensitivity of reproductive organs is currently of great interest for increasing seed set. The responses of seed set to HT involve multiple processes in both male and female reproductive organs, but we currently lack an integrated and systematic summary of these responses for the world's three leading food crops (rice, wheat, and maize). In the present work, we define the critical high temperature thresholds for seed set in rice (37.2°C ± 0.2°C), wheat (27.3°C ± 0.5°C), and maize (37.9°C ± 0.4°C) during flowering. We assess the HT sensitivity of these three cereals from the microspore stage to the lag period, including effects of HT on flowering dynamics, floret growth and development, pollination, and fertilization. Our review synthesizes existing knowledge about the effects of HT stress on spikelet opening, anther dehiscence, pollen shedding number, pollen viability, pistil and stigma function, pollen germination on the stigma, and pollen tube elongation. HT-induced spikelet closure and arrest of pollen tube elongation have a catastrophic effect on pollination and fertilization in maize. Rice benefits from pollination under HT stress owing to bottom anther dehiscence and cleistogamy. Cleistogamy and secondary spikelet opening increase the probability of pollination success in wheat under HT stress. However, cereal crops themselves also have protective measures under HT stress. Lower canopy/tissue temperatures compared with air temperatures indicate that cereal crops, especially rice, can partly protect themselves from heat damage. In maize, husk leaves reduce inner ear temperature by about 5°C compared with outer ear temperature, thereby protecting the later phases of pollen tube growth and fertilization processes. These findings have important implications for accurate modeling, optimized crop management, and breeding of new varieties to cope with HT stress in the most important staple crops.

Key words: high temperature, seed set, floret organs, flowering pattern, pollination

Liu M., Zhou Y., Sun J., Mao F., Yao Q., Li B., Wang Y., Gao Y., Dong X., Liao S., Wang P., and Huang S. (2023). From the floret to the canopy: High temperature tolerance during flowering. Plant Comm. **4**, 100629.

INTRODUCTION

Extreme hot weather induced by climate change has become increasingly serious in its frequency, intensity, and duration (Stocker et al., 2014). Heat waves, periods of extreme high temperature that usually last for several days or even weeks, greatly threaten global agricultural production (Chakraborty et al., 2019). In major countries for maize (*Zea mays* L.), rice (*Oryza sativa* L.), and wheat (*Triticum aestivum* L.) production worldwide, high-temperature (HT) stress occurs frequently during the life cycle of these crops (Supplemental Figures 1A–1C; Gourdji et al., 2013; Hassan et al., 2020). If the global mean temperature increases by 1°C without CO_2 fertilization, the warmer temperature is estimated to reduce crop yield by 7.4% in maize, 3.2% in rice, and 6% in wheat (Zhao et al., 2017). The global

Published by the Plant Communications Shanghai Editorial Office in association with Cell Press, an imprint of Elsevier Inc., on behalf of CSPB and CEMPS, CAS.

This is an open access article under the CC BY license (http://creativecommons.org/licenses/by/4.0/).

average near-surface temperature has increased by 0.8°C with a more rapid speed in the past 30 years compared with 1961–1990 (Supplemental Figure 1D; Morice et al., 2021). Yield loss is expected to be exacerbated if heat waves occur at the flowering stage, as this is the most heat-sensitive period for successful sexual reproduction and grain yield formation (Hedhly et al., 2009; Lohani et al., 2020; Xu et al., 2020; Impa et al., 2021).

The rapid increase in global nighttime temperature, which has been rising 1.4 times faster than daytime temperature over the past five decades (Solomon et al., 2007), makes nighttime heat stress another threat to food production (Coast et al., 2015; García et al., 2015; Wang et al., 2020b; Sakai et al., 2022). Although nighttime heat stress during flowering has been shown to negatively impact spikelet fertility, its effects are less pronounced under controlled and field conditions (Shi et al., 2013; García et al., 2015; Jagadish et al., 2015; Wang et al., 2020b). Hence, our focus is mainly on how daytime heat stress during flowering effects grain number formation in the three major staple cereals, which together contribute more than 85% of global grain production.

Successful pollination and seed set are dependent on crop male and female reproductive organs in relation to growth and development, flowering pattern, reproductive organ activity, and the fertilization process (Jagadish, 2020; Wang et al., 2021a; Zhu et al., 2021). These processes are sensitive to HT stress, even for short periods of time (Hedhly et al., 2009; Prasad et al., 2017; Lohani et al., 2020). Heat stress for 2–5 days around flowering significantly reduced seed set in rice (pre- or post-heading stage; Endo et al., 2009; Fu et al., 2016) and maize (pre- and post-silking; Wang et al., 2020a, 2020b), resulting in irreversible yield losses (Zhu et al., 2021). HT-induced yield losses are partly attributable to disruption of flowering behaviors, including an extended anthesis-silking interval in maize and a small spikelet opening angle, reduced pollen shedding number due to failure of anther dehiscence, and early morning flowering, night flowering, or lack of flowering in wheat (Steinmeyer et al., 2013; Aiqing et al., 2018; Wang et al., 2019b; Matsui and Hasegawa, 2019; Chen et al., 2020). In addition, HT stress during flowering can negatively affect the function of male and female reproductive organs, reducing floret fertility and ultimately lowering seed number (Hays et al., 2007; Arshad et al., 2017; Smith, 2019). Rice, wheat, and maize have different HT thresholds at flowering, beyond which seed set and grain number will be significantly reduced (Sanchez et al., 2014), suggesting that these three cereals likely respond differently to HT stress. As a monecious crop, maize has individual male (tassel) and female (ear) flowers on the same plant, with the tassel exposed directly to sunlight. Wheat and rice plants have bisexual flowers, with several florets in one spikelet of wheat and a single floret in each fertile spikelet of rice (Bortiri and Hake, 2007; Gol et al., 2017). Unlike wheat, maize and rice experience more HT events in space and time, although temperatures in the paddy field are expected to be more stable for rice growth (Alberto et al., 2009; Matsui et al., 2021). As a tall crop with a high canopy structure, maize creates a more favorable microenvironment for ear growth and fertilization (Khush and Peng, 1998; Pangga et al., 2013; Tivoli et al., 2013). The maize canopy has been shown to produce a temperature difference of up to 8.5°C between ears directly exposed to sunlight and those protected by the canopy (Khabba et al., 2001). Furthermore, the husk leaves of maize ears can reduce the inner ear temperature by 5°C compared with the outer ear temperature (Wang et al., 2023), alleviating the negative effects of HT stress on pollen tube growth, fertilization, and zygote development (Salvador and Pearce, 1988; Khabba et al., 1999, 2001; Cui et al., 2020). These differences in growing environments, phenology, morphology, and physiology produce the common and unique mechanisms by which maize, rice, and wheat cope with HT stress through resistance and adaptation. Despite its importance, this information has yet to be systematically synthesized.

In this review, our objective was to comprehensively assess the effects of HT stress on seed set, flowering, and fertilization of rice, wheat, and maize. This involved (1) determining the critical HT thresholds of these crops for seed set, floret growth and development, and pollination and fertilization; (2) evaluating their sensitivities to HT stress at key developmental stages during flowering; (3) analyzing the effects of HT stress from the ecosystem on reproductive organs and tissues; (4) synthesizing existing knowledge about the adaptation and escape mechanisms of crops to HT stress; and (5) summarizing the current status of research on HT stress around flowering. We hope that research on accurate modeling, crop management optimization, and variety breeding strategies associated with HT stress during flowering will benefit from this study.

Sensitivity of seed set to HT stress around flowering in maize, rice, and wheat

Critical temperature thresholds for seed set

We collected data from experiments with accurately and/or non-accurately controlled environments (e.g., growth cabinet/chamber, sun-lit phytotron, and field-based heat tents), yielding a total of 43 articles: 8 for maize, 25 for rice, and 10 for wheat. Based on a fitted curve of a non-linear Boltzmann model to all measured values, we estimated the daytime and nighttime HT thresholds for seed set during flowering for each crop (Figure 1; Supplemental Table 1; Wang et al., 2021b). In this study, HT thresholds during flowering are defined as the temperatures above which seed set will be significantly reduced. Our findings estimated the high daytime temperature (HDT) thresholds (with standard error [SE]) to be 37.9°C (SE 0.4°C; n = 202) in maize, 37.2°C (SE 0.2°C; n = 386) in rice, and 27.3°C (SE 0.5°C; n = 202) in wheat (Figure 1A). Daytime temperatures whose average exceeds the critical threshold across hourly, daily, and weekly time courses overlapping with the crop reproductive period cause highly variable reductions in seed set (Supplemental Table 2). Heat waves that last for more than 1 week cause the seed set of rice, wheat, and maize to fall below 60%. Our results indicated that maize and rice had a similar sensitivity to the stress of HDT at anthesis. The temperature threshold of 37.9°C for anthesis in maize was confirmed in controlled environment experiments in the study of Wang et al. (2021b), who used six temperature levels ranging from 30°C/20°C to 40°C/30°C at 2°C increments over nearly 2 weeks bracketing the silking stage. In this study, seed set of two maize hybrids ranged from 20% to 50% after heat treatment of >38°C that lasted for 2 weeks. In rice, a similar temperature threshold of 37°C (SE 1.2°C) at anthesis was defined by Sanchez et al. (2014), with *japonica* varieties

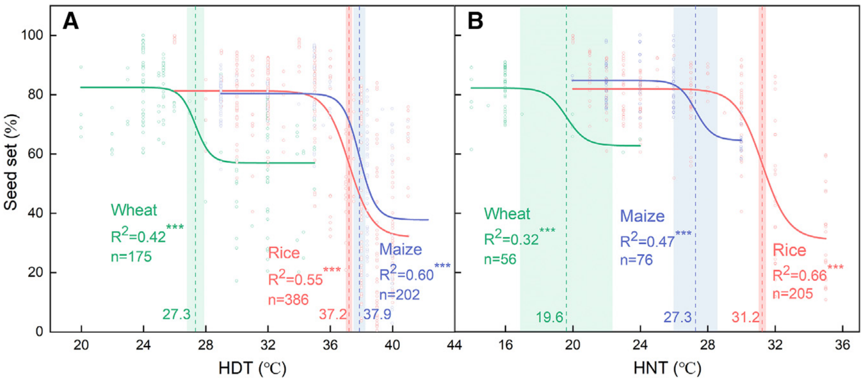

Figure 1. Response of seed set (%) in three major global cereal crops (rice, wheat, and maize) to high day temperature (HDT; A, °C) and high night temperature (HNT, B, °C) during the flowering stage.
The curves are based on measured values obtained from the literature and fitted using the nonlinear Boltzmann model. Each fit line in the figure is successful, and the number of data points (*n*) used in each fit and the coefficient values (R^2) with significance labels (*) are shown. The vertical dashed lines indicate the critical temperature thresholds that lead to significant changes in seed set. The shaded area denotes the standard error (SE) calculated from the standard deviation of the compiled data used to estimate the mean. More detailed model parameters are provided in Supplemental Table 1. Crops and related references: rice – Matsui et al., 2001; Matsui and Omasa, 2002; Matsui and Hasegawa, 2019; Cheng et al., 2009; Endo et al., 2009; Mohammed and Tarpley, 2009, 2010, 2011; Mohammed et al., 2013; Ishimaru et al., 2010; Jagadish et al., 2010, 2015; Madan et al., 2012; Shi et al., 2013, 2016, 2017; Zhang et al., 2013; Coast et al., 2015; Hirabayashi et al., 2015; Koike et al., 2015; Fu et al., 2016; Wu et al., 2016, 2019; Yan et al., 2017; Chen et al., 2020; wheat – Jäger et al., 2008; Prasad et al., 2008a, 2008b, 2011; Khan et al., 2015; Narayanan et al., 2015; Joshi et al., 2016; Aiqing et al., 2018; Bheemanahalli et al., 2019; Fábián et al., 2019; Djanaguiraman et al., 2020); and maize – Yu et al., 2016; Li et al., 2019; Wang et al., 2019, 2020a, 2020b, 2021a, 2021b; Sheng et al., 2020; Mu et al., 2022; Liu et al., 2023.

having a lower threshold of 36.9°C (SE 2.2°C). Several temperature-controlled trials have shown that rice spikelet fertility was reduced by >50% when HT (>37°C) lasted for less than 1 week at the middle stage of heading (Matsui et al., 2001, Matsui and Omasa, 2002; Jagadish et al., 2007; Chen et al., 2020). The SE is higher in maize than in rice, perhaps because maize is grown at a wider range of altitudes and latitudes, ranging from ca. 60°N to 40°S, including both cool and hot regions (Shiferaw et al., 2011). The high SE may also be due to variability in HT tolerance among varieties and in experimental designs among studies.

Interestingly, maize had a lower high nighttime temperature (HNT) threshold during flowering than did rice (27.3°C vs. 31.2°C; Figure 1B; Supplemental Table 1), making maize more sensitive than rice to HNT during flowering. When nighttime temperature exceeds the critical threshold for more than 1 week, seed set of rice and maize generally remains at 30%–60%, depending on crop type and actual temperature levels (Supplemental Table 2). With climate warming, nighttime temperature is rising 1.4 times faster than daytime temperature, and this has a negative impact on crop grain yield (Peng et al., 2004; Solomon et al., 2007; Sadok and Jagadish, 2020; Impa et al., 2021). In the study of Peng et al., (2004), rising nighttime temperature was expected to pose larger threats to grain yield in rice than daytime temperature, as grain yield declined by 10% with a 1°C increase in growing season nighttime temperature. HNT during flowering, even for a short period, can induce spikelet sterility and directly reduce grain yield in both rice and maize by altering flowering patterns, reducing pollen viability, and increasing spikelet degeneration (Laza et al., 2015; Wang et al., 2020b; Sakai et al., 2022). When HDT stress is followed by HNT stress, the negative effects of HDT on flowering events can be greatly exacerbated by HNT (Sakai et al., 2022). Even if plants attempt to avoid HDT stress bracketing the midday by rescheduling their flowering (Ishimaru et al., 2010; Jagadish et al., 2015), they are unable to escape stress damage caused by elevated nighttime minimum temperature (Sakai et al., 2022). Under HNT conditions, leaf respiration increases, reducing the carbon available for plant growth and yield formation (Cantarero et al., 1999; Laza et al., 2015). In addition, reactive oxygen species (ROS) accumulate rapidly in reproductive organs, directly effecting pollination and lowering seed set (Sadok and Jagadish, 2020; Impa et al., 2021). Furthermore, HNT increases night vapor pressure deficit (VPD), a combination of temperature and relative air humidity, which slows plant growth by reducing water content in plant tissues (Turc et al., 2016; Grossiord et al., 2020). It is noteworthy that both vegetative and reproductive organs grow more rapidly in the nighttime than in the daytime (Zweifel et al., 2021), and increased night VPD is expected to disturb flowering patterns (Turc et al., 2016). Presumably, the higher HNT sensitivity at flowering in maize is associated with suppression of silk growth and reduction in silk emergence rate as a result of high nighttime VPD and temperature.

Compared with maize and rice, wheat exhibits lower HDT (27.3°C) and HNT (19.6°C) thresholds for anthesis (Figure 1B), which reflects a greater susceptibility of wheat reproductive events to HT stress. Either HDT or HNT that lasts for more than 1 week can reduce seed set of wheat to about 60% (Supplemental Table 2). The vulnerable anthesis of wheat sometimes meets with HTs above 27°C in European regions (Semenov and Shewry, 2011; Stratonovitch and Semenov, 2015), and the frequency of such events is increasing with the warming climate. Porter and Gawith (1999) found that the critical temperature threshold for loss of wheat grain number due to pollen sterility was 31°C (SE 3.7°C) at anthesis, which was 3.7°C higher than the temperature estimated in the present study. A temperature threshold of 31°C was identified based on HT shortly before anthesis (~5 days) in the study of Wheeler et al. (1996a,1996b) based on limited compiled data (*n* = 1; Porter and Gawith, 1999). Wheeler et al. (1996a) and Mitchell et al. (1993) also found that a daytime temperature of 27°C or more that lasted for more than a week at 50% anthesis resulted in a high proportion of wheat grain abortion and substantial yield losses; this estimate was very close to the estimated threshold of 27.3°C in our study. Stone and Nicolas (1995)

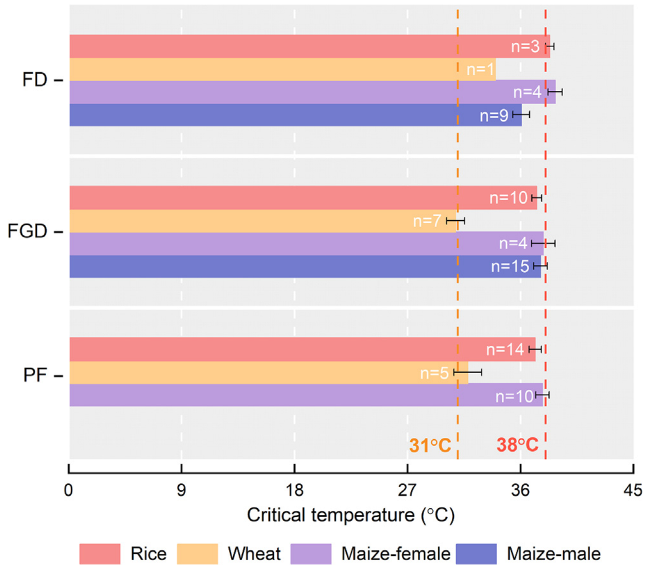

Figure 2. Figure 2Critical temperatures for key flowering traits of three major cereal crops around the flowering stage.
FD, flowering dynamics—flowering time, peak, and duration for rice and wheat, tasseling and shedding pollen time, date, and duration for maize male flowers (tassel), and silk emergence date and duration for maize female flowers (ear); FGD, floret growth and development—spikelet opening, anther dehiscence, stigma/silk elongation, pollen production, and/or silk emergence rate for rice, wheat, and maize florets; PF, pollination and fertilization—pollen and stigma spatiotemporal interaction, pollen germination, pollen tube elongation, and double fertilization for female reproductive organs. Compared with rice and wheat, maize has separate critical temperatures for FD and FDM because maize has separate male and female organs. Temperature data were obtained from the articles and averaged as the critical temperature (i.e., mean ± SE) beyond which crop flowers were injured, flowering behavior/habit changed, or physiological processes ceased. The "*n*" values represent the number of temperature data points collected for the traits from the articles. All references are listed in the supplemental information.

revealed that plants were most sensitive to HDT in the first 3 days after anthesis in Australian wheat varieties. An HNT threshold of 19.6°C (SE 2.7°C) for anthesis was also identified in wheat (Figure 1B), consistent with the findings of Prasad et al. (2008a, 2008b), who reported that wheat spikelet fertility and grains per spike were significantly lower when nighttime temperatures exceeded 20°C. These results indicate that the temperature threshold may vary over a daily or weekly time course at anthesis. The temperature thresholds estimated in our study were based on experimental results for short time courses before, after, and bracketing the anthesis stage in different varieties, providing a comprehensive understanding of temperature limitations on wheat seed set during flowering.

Key flowering traits and their respective critical temperatures

In most studies of HT stress, reductions in grain yield and spikelet/floret fertility in maize, rice, and wheat have frequently been attributed to negative effects of HT on flowering dynamics, floret growth and development, and pollination and fertilization (PF) (Supplemental Table 3). Using the critical temperature assessment method of Sanchez et al. (2014), we estimated the critical temperatures for key flowering traits of the three main cereal crops around the flowering stage (Figure 2). Critical temperatures for anthesis were estimated to be 38°C for rice and 34°C for wheat. Above these temperatures, each increase of 1°C caused significant changes in the onset, peak, and/or duration of anthesis, resulting in 2.4%–7% spikelet fertility loss in rice and 22%–38% grain yield loss in wheat (Jagadish et al., 2007; Aiqing et al., 2018). Rice and wheat plants can partially adjust daily flowering patterns, for example, by moving the peak of flowering toward cooler early morning or evening, in an attempt to reduce the effects of HT stress on seed set (Bheemanahalli et al., 2017; Aiqing et al., 2018). For monoecious and cross-pollinated crops, maize plants bear separate male and female flowers, and HTs above 36°C significantly advance tasseling and pollen shedding time (male reproductive organs; Wang et al., 2019b). HT stress has slight effects on female reproductive organs in maize, but when temperature increased to 38°C, silking time was significantly delayed, extending the anthesis-silking interval and increasing kernel loss (Edreira et al., 2011; Wang et al., 2021b; Liu et al., 2022b). This creates complexity for understanding the effects of HTs on maize yield formation, as male and female reproductive organs differ in their critical temperatures at flowering. Temperatures in excess of 38°C in rice and maize or 31°C in wheat during floret development can cause irreversible damage to PF (Figure 2; Supplemental Table 3) due to changes in lodicule expansion, anther dehiscence, pollen shedding and germination, pollen tube growth, and style growth and activity (Saini and Aspinall, 1982; Matsui et al., 2000; Prasad and Djanaguiraman, 2014; Begcy et al., 2019; Matsui and Hasegawa, 2019; Chen et al., 2020). Because of the difficulty in identifying critical temperatures for PF, especially in rice and wheat, the critical temperatures for pollen germination, pollen tube elongation, and/or zygote formation were averaged in this study. The critical temperature for PF was estimated to be 37.8°C ± 0.5°C in maize, followed by 37.2°C ± 0.5°C in rice and 31.8°C ± 1.1°C in wheat (Figure 2), similar to the order of the HDT thresholds for seed set (Figure 1A). In brief, the "penalty" mechanism by which HT stress above the HT threshold affects crop yield is primarily a "derailing" of normal flowering events.

Temperature sensitivity at different phases around flowering

In crops, two stages are particularly sensitive to HT stress: (1) floral bud development during micro- or megasporogenesis and (2) the flowering stage (Prasad et al., 2001; Prasad et al., 2008a; Wassmann et al., 2009). In maize, silk emergence is frequently regarded as an important index and time step for evaluating the effects of HT stress on flowering pattern and seed set (Cárcova et al., 2003; Borrás et al., 2007; Liu et al., 2022a). Results from field experiments indicated that 2 weeks of HT stress after silking had greater effects on kernel number per ear than pre-silking HT stress (Dong et al., 2021; Liu et al., 2022b). In a controlled environment experiment, 5 days of pre-silking HT stress (40°C/30°C) reduced maize seed set by ~10% compared with that of the respective control (32°C/22°C), whereas 5-day post-silking HT stress reduced seed set by ~23% (Figure 3A; Wang et al., 2020a). A post-pollination heat spell over an hourly time course resulted in pollination failure by derailing pollen tube growth in maize silk (Dupuis and Dumas, 1990). This evidence reveals that the post-silking growth stage (e.g.,

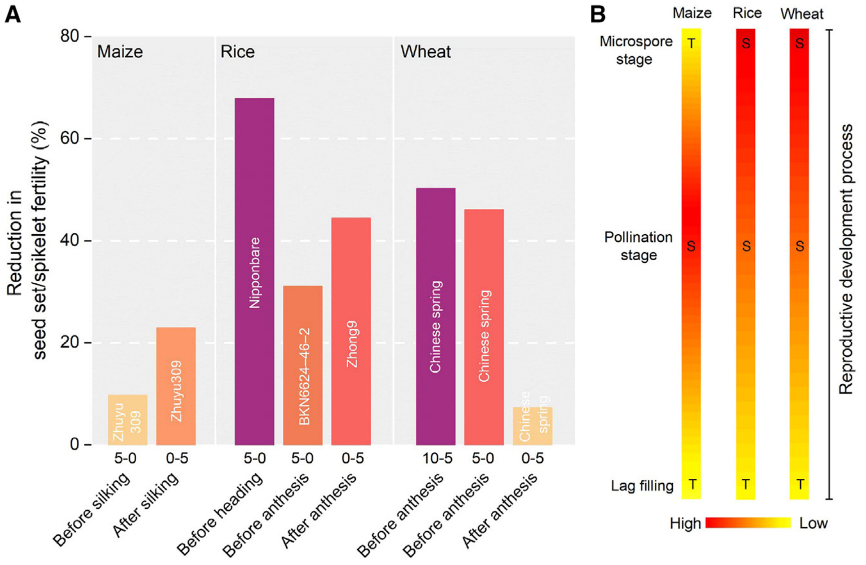

Figure 3. Reduction in seed set/spikelet fertility relative to normal conditions for maize, rice, and wheat under high-temperature (HT) stress around flowering (A) and their temperature sensitivity at different phases around flowering (B).
Detailed information related to the data: (1) maize, 40°C/30°C for HT stress treatment (day/night temperature) and 32°C/22°C for normal temperature treatment occurred 5–0 days before silking and 0–5 days after silking (Wang et al., 2020a, 2020b); (2) rice, 39°C/30°C for HT stress treatment and 28°C/22°C for normal temperature treatment occurred 5–0 days before heading (Endo et al., 2009); 35°C air temperature–induced heat injury occurred during different stages of panicle development for 5 days, and 5 days of HT before anthesis produced the highest reduction in spikelet fertility (Yoshida et al., 1981; Wassmann et al., 2009); HT stress of 40°C/30°C and non-stressed temperatures of 33°C/24°C were applied to a heat-sensitive rice plant (Zhong9) for 5 days from anthesis (Fu et al., 2016); (3) wheat, 36°C/26°C for HT stress treatment and 25°C/15°C for normal temperature treatment occurred at 10–5 and 5–0 days before anthesis and 0–5 days after anthesis (Prasad and Djanaguiraman, 2014). The white words on the bars of the graph **(A)** are the names of varieties chosen in the above literature. Based on the reduction in seed set/spikelet fertility (%) of maize, rice, and wheat under different HT stress treatments and referring to Jagadish (2020), we inferred HT stress sensitivity/tolerance of reproductive developmental processes (from the microspore stage to the lag period) in maize, rice, and wheat **(B)**.

pollination) is most sensitive to HT stress during maize kernel formation.

Unlike maize, rice and wheat experienced greater kernel abortion in response to pre-heading HT stress rather than post-anthesis HT stress (Figure 3A). Evidence from studies in rice indicated that 5–0 days of HT stress (39°C–40°C) before heading caused greater reductions in seed set than did post-anthesis HT relative to their respective control treatment (~70% vs. ~45%; Endo et al., 2009; Fu et al., 2016). In addition, heat damage induced by 35°C air temperature 5–0 days before anthesis resulted in ~30% reduction in spikelet fertility (Yoshida et al., 1981; Wassmann et al., 2009). In wheat, two phases (8–6 days and 2–0 days before anthesis) were found to be more sensitive to short episodes (2–5 days) of HT stress. These results suggest that aspects of early floret development such as microspore development in rice and wheat are most sensitive to HT (Figure 3B), mainly because of effects on pollen viability (Endo et al., 2009; Prasad and Djanaguiraman, 2014; Bheemanahalli et al., 2019). HT stress during microsporogenesis can dramatically reduce pollen viability by affecting basic metabolic pathways such as starch, lipid, and energy biosynthesis (Prasad and Djanaguiraman, 2014; Djanaguiraman et al., 2018; Begcy et al., 2019) and by arresting anther development (Abiko et al., 2005; Sakata and Higashitani, 2008).

In summary, stage-dependent sensitivities to HT stress in cereal crops show both similarities and differences among rice, wheat, and maize. The microsporogenesis stage, particularly the tetrad stage, is highly sensitive to HT stress across all three cereals (Wassmann et al., 2009; Barber et al., 2017; Begcy et al., 2018, 2019; Smith, 2019). However, in maize, the post-silking growth stage in the daily time course appears to be more sensitive to HT stress than the microsporogenesis stage (Wang et al., 2020a), as multiple flowering events occur simultaneously in the short post-silking period, including spikelet opening, anther dehiscence, pollen shedding, silking, pollen germination, pollen tube elongation, double fertilization, and early embryo formation (De Jong and Klinkhamer, 2005; Tranel, 2007; Riechmann and Wellmer, 2014).

Floret characteristics and flowering behaviors under HT stress

Floret characteristics

In rice, each spikelet contains one fertile floret, two sterile lemmas, and two rudimentary glumes, with each floret consisting of six stamens, one pistil, and two lodicules enclosed by the lemma and palea (Supplemental Figures 2A and 2E). In wheat, the spikelet meristem (SM) initiates several floret meristems (FMs) and florets on each spike rachis node (corresponding to 2–3 grains in one spikelet), each of which is enclosed by a pair of glumes and consists of three stamens, one pistil, one lemma, one palea, and two lodicules (Supplemental Figures 2B and 2F; Gol et al., 2017). In maize, male flowers are located on the tassel and female flowers on the ear (Supplemental Figures 2C, 2D, 2G, and 2H). The floret in the tassels consists of three stamens, one lemma, one palea, and two lodicules (Dellaporta and Calderon-Urrea, 1994; Tranel, 2007). In the ear, each spikelet produces two FMs (upper and lower FMs), although only the upper FM normally develops into a floret (Bortiri and Hake, 2007). The floret organs in the ear are wrapped by leaf sheath and husk leaves.

The distinct morphological features of spikelets and florets in maize, rice, and wheat probably contribute to their varying levels of tolerance to HT stress during flowering. The protective structures, such as glumes, lemma, palea, or husks in the maize ear, shield the reproductive organs (e.g., stamens and/or pistil) from the negative effects of HT stress. For example, cleistogamy in

rice, in which the florets remain closed during anthesis, has been shown to reduce the negative effects of HT stress (>38°C) during flowering on seed set by providing an internal environment with a temperature that is approximately 1.8°C lower than the outside temperature (Koike et al., 2015). Reduced exposure of the stigma within glumes to HT stress has also been reported to improve spikelet fertility and heat tolerance (Wu et al., 2019). In addition, the sheathed panicle phenotype in rice was reported to ensure sufficient pollen number and germination on the stigma for successful pollination at higher spikelet tissue temperatures (38°C–40°C) at anthesis, and total spikelet number was thus unaffected by heat stress (Lawas et al., 2018).

In maize tassels, the glumes of the spikelet and the lemma and palea of the floret are designed to open at anthesis, promoting successful pollination but also likely decreasing the spikelet's tolerance to HT stress during male gametophyte development (Mitchell and Petolino, 1988). The maize ear is located in the middle section of the plant and is wrapped by multiple layers of husk leaves, which regulate the temperature inside the ear to be 2°C–3°C lower than the outside temperature (Khabba et al., 2001; Wang et al., 2023). However, HTs above 38°C can delay silk emergence, reduce silk emergence rate, and greatly limit seed set (Liu et al., 2022a). Compared with research on the tassel (male floret), there has been less focus on the effects of HT on female floret development and fertility in maize.

Flowering behaviors

HT stress can advance, delay, or inhibit flowering in cereal crops, depending on its severity (Cicchino et al., 2010; Arshad et al., 2017; Aiqing et al., 2018; Wang et al., 2021b). HTs at 36°C advanced tasseling and anthesis in maize (Wang et al., 2019b), whereas HTs >38°C induced spikelet closure in rice (Supplemental Figure 3D; Wang et al., 1989; Jagadish et al., 2007; Yan et al., 2017; Yang et al., 2020). In cereal crops, spikelet opening is the result of floret opening and anther extrusion, which is rather short and usually lasts for less than 30 min (Supplemental Figures 3A–3C; De Vries, 1971; Ishimaru et al., 2012; Yan et al., 2017). The swelling of lodicules located between the lemma and the ovary base drives floret and spikelet opening by pushing away the rigid lemma and palea, which is an essential pre-condition for pollination in maize and cross-pollination in rice and wheat (Heslop-Harrison and Heslop-Harrison, 1996; Qin et al., 2005; Yoshida, 2012; Beauzamy et al., 2014; Xiao et al., 2014). In wheat, the ability to disperse pollen for cross-pollination depends on anther extrusion after spikelet opening (Denisow et al., 2022). A secondary opening in wheat florets, which follows the lodicule-induced opening that results from lateral expansion of unfertilized ovaries, increased seed set via cross-pollination (Okada et al., 2018). Silk (stigma) emergence from the husk is the other half of the flowering event in maize, lasting for nearly 20 days from the first silk elongation from basal ovules of the cob (ca. 10–14 days prior to the silking stage, corresponding to the ~12-leaf stage; Nielsen, 2016). This prolonged period increases the likelihood that silk elongation and emergence will meet with abiotic stress, thus reducing silk viability and emergence rate (Oury et al., 2016; Liu et al., 2022a), although this has rarely been investigated compared with tassel flowering patterns.

The lodicule as the first "switch" for floret opening in cereal crops has been an increasing focus of abiotic stress research (Figure 4A–4C; Liu et al., 2017; Chen et al., 2020; Yang et al., 2020). The swelling of lodicules is dependent on osmotic regulation substances (ORSs) and water potential (Figure 4D; Honda et al., 2005; Liu et al., 2017). Studies in rice confirmed that HT-induced spikelet closure, even in a short period of 45 h, was mainly attributable to failed swelling of lodicules (Supplemental Figures 3D and 3G; Chen et al., 2020). Jasmonic acid (JA) in lodicules has been found to play a role in their water uptake and release, and its regulation of swelling and withering under HT stress has been verified by molecular evidence (Cai et al., 2014; Xiao et al., 2014; Yan et al., 2017; Yang et al., 2020). The gene *OsJAR1*, which encodes JA-amino acid synthetases such as JA-isoleucine synthase and is essential for controlling the timing of floret opening, has been identified as a key member of the JA signaling pathway in the lodicule (Xiao et al., 2014). In *osjar1* mutants in which *OsJAR1* is silenced, the lodicule K^+ concentration is higher than that of the wild type, suggesting a role for K^+ homeostasis in regulation of lodicule swelling and withering (Chen et al., 2016). Liu et al. (2017) found that JA deficiency reduced lodicule swelling mainly by retarding the accumulation of ORSs such as soluble sugars through downregulation of *OsAOC* expression. Application of methyl jasmonate has been shown to induce floret opening and increase the numbers of opening florets, thus increasing tolerance to HT stress during flowering (Zeng et al., 1999; Yan et al., 2017; Yang et al., 2020). In addition, an inward calcium (Ca^{2+}) flux across the plasma membrane is considered to be the primary pathway for heat signal transduction under HT stress (Mittler et al., 2012), strengthening the integrity of cell membranes and stimulating the synthesis of JA (Wang et al., 2019a; Gao et al., 2019). The plant steroid hormones brassinosteroids (BRs) participate in regulating lodicule swelling by affecting the accumulation of ORSs upon HT stress during flowering in relation to the expression of *sterol methyltransferase2* (Figure 4D; Liu et al., 2023). *Sterol methyltransferase2* encodes sterol 24-carbon methyltransferases that are intermediates in BR biosynthesis (Fujioka and Yokota, 2003). Exogenous epibrassinolide increased the spikelet opening angle and number of opening spikelets in the maize tassel, confirming the ability of BRs to enhance tassel flowering (Liu et al., 2023).

Vascular bundles in the lodicule are involved in water uptake and release (Wang et al., 1991). Evidence from maize and rice indicated that a large number of vascular bundles in the lodicule is unfavorable for the persistence of spikelet opening (Yan et al., 2017; Liu et al., 2023) because water release occurs mainly from the vascular bundles of the lodicules rather than from the lodicule surface (Wang et al., 1991). HT stress can accelerate water release from the lodicules via vascular bundles and thus inhibit spikelet opening or promote spikelet closing.

Heat stress is on: Pollen release, pollen germination, and pollen tube elongation

Anther dehiscence and pollen release

Anther dehiscence, followed by spikelet opening, is the second "switch" for pollen release (Figure 4E; Keijzer et al., 1996; Wilson et al., 2011), but it is highly sensitive to HT stress (Supplemental Figure 3H; Giorno et al., 2013; Khan et al., 2020; Zhang et al., 2021). HT stress, even for a single day, can impact

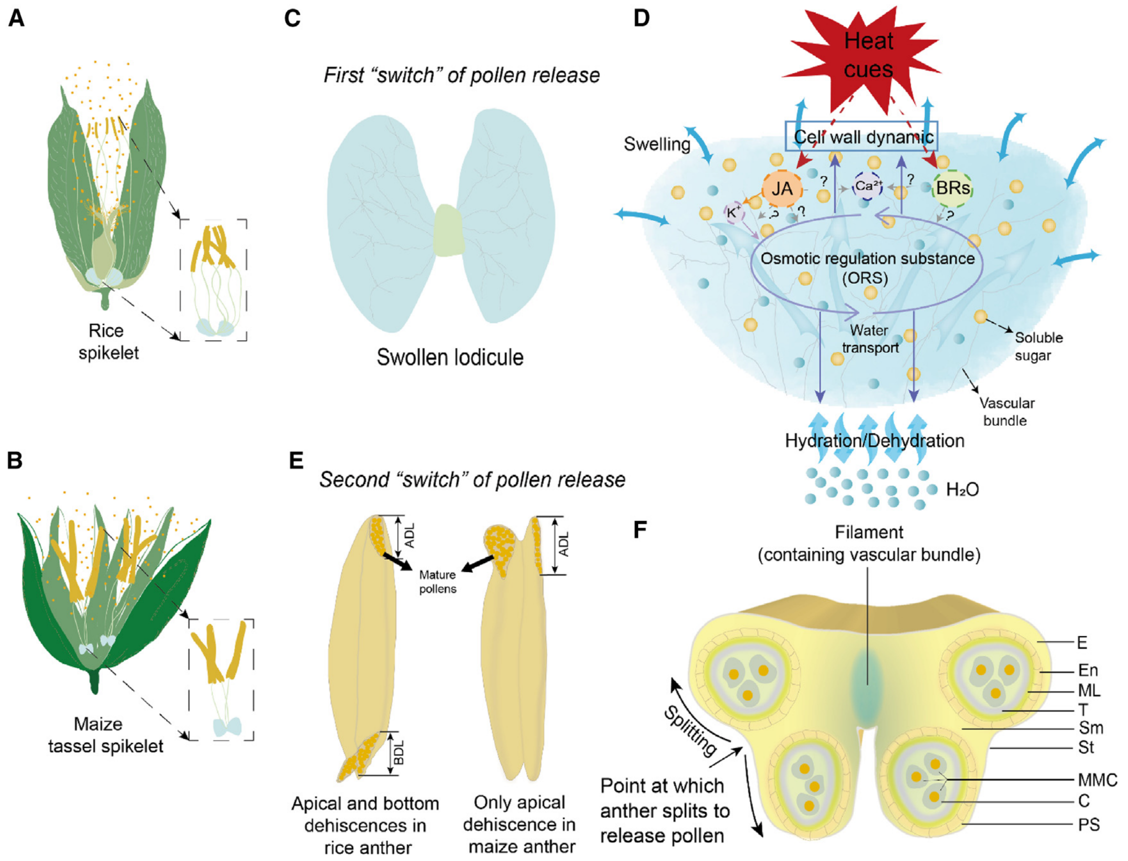

Figure 4. Simplified model of the lodicule swelling mechanism, anther dehiscence types, and anther cross-sectional structure of rice and maize spikelets.

The completion of spikelet-opening behavior as the first "switch" for pollen release in rice and maize during flowering requires the driving force provided by water absorption and swelling of the lodicule **(A–C)**. HT stress disrupts hormone synthesis, signaling regulation (JA and BRs in both rice and maize), and downstream targets (i.e., K^+, Ca^{2+}), leading to an imbalance in osmotic regulation substances (ORSs) in the lodicules. A large vascular bundle system can accelerate water loss from the lodicule under heat stress **(D)**. Anther dehiscence as the second "switch" for pollen release occurs in two different types: apical and bottom dehiscence occur in the rice anther, but only apical dehiscence occurs in the maize anther **(E)**. **(F)** illustrates the cross-sectional morphology of anther development, with four pollen sacs consisting of the epidermis, endothecium, middle layer, tapetum, and reproductive cell layer, modified from Zhang et al. (2021). The microspore mother cells first undergo meiosis to form tetrads wrapped by callose and then proceed with mitoses to produce mature pollen grains. In the later stage of anther development, the septum and stomium gradually dehisce at the point where the anther splits to release pollen grains. JA, jasmonic acid; BRs, brassinosteroids; K^+, potassium; Ca^{2+}, calcium; ADL, apical dehiscence length; BDL, bottom dehiscence length; E, epidermis; En, endothecium; ML, middle layer; T, tapetum; Sm, septum; St, stomium; MMC, microspore mother cells; C, callose; PS, pollen sac.

anther structure and pollen wall morphology (Hedhly et al., 2009). The anther is comprised of four pollen sacs, each with four cell layers: the epidermis, endothecium, middle cell layers, and a secretory tapetum that surrounds the inner sporophytic cells (Figure 4F; Scott et al., 2004; Wilson et al., 2011). Anther opening is a function of localized cellular differentiation and degeneration as well as changes in the structure and water status of the anther, including enzymatic breakdown of the septum, programmed cell death (PCD) of the septum, stomium, and endothecium, secondary thickening, pollen swelling, and anther dehydration (Kuriyama and Fukuda, 2002; Sanders et al., 2005; Wilson et al., 2011). Phytohormones, particularly JA and auxin (IAA), play important roles in regulating anther dehiscence, filament elongation, and pollen viability (Scott et al., 2004; Cecchetti et al., 2008). Deficiencies in JA can delay or prevent anther dehiscence because JA is involved in tapetal degeneration, septum breakdown, and endothecium thickening (Ishiguro et al., 2001). Local increases in auxin can also delay anther dehiscence by hindering septum breakdown (Yasuor et al., 2006; Cecchetti et al., 2008). Ethylene defects also retard anther dehiscence by delaying degeneration of the stomium cells and dehydration (Rieu et al., 2003). Gibberellins are involved in the regulation of tapetal breakdown and the initiation of dehiscence and hence regulate anther dehiscence (Hu et al., 2008). In addition, the *OsHXK10* promoter was found to drive the expression of β-glucuronidase specifically in anther wall, and its downregulation inhibited anther cell wall thickening and resulted in non-dehiscence (Xu et al., 2008).

HT stress affects anther dehiscence by inhibiting tapetum differentiation and microsporogenesis and promoting the PCD process in anther cells, especially in the endothecium layer, and it thus results in male sterility (Kim et al., 2001; Giorno et al., 2013; Zhang et al., 2021). Under HT stress conditions, levels of JA, IAA, and

gibberellins are reduced, whereas abscisic acid (ABA) levels increase in the anther, leading to reduced spikelet fertility (Tang et al., 2007; Khan et al., 2022a). Reduced JA levels impede the thickening of the secondary wall in endothecium cells by reducing lignification and result in indehiscent anthers due to accumulation of excessive ROS in anthers (Khan et al., 2022a). HT-induced ROS accumulation has been observed in the anthers of many crops (Djanaguiraman et al., 2018), and disruption of ROS homeostasis can result in undesirable PCD of the endothecium cells (Khan et al., 2022b). In rice, higher ABA levels and ABA-induced ROS accumulation in HT-stressed anthers resulted in earlier initiation of PCD induction and subsequently in abnormal tapetum degeneration (Bheemanahalli et al., 2020; Zhao et al., 2023). SAPK2 is required for ABA-induced ROS generation in the developing anther, but reduced ROS accumulation due to impaired ABA signaling was not observed in heat-stressed anthers of the *OsSARK2* knockout mutant (Zhao et al., 2023). HT stress has direct effects on anther development and results in malformed structures, including a knitted anther cuticle structure of the epidermis, an undegraded septum, a thickened anther wall, and unevenly distributed Ubisch bodies (Hu et al., 2021). In addition, HT stress during flowering can also inhibit swelling of pollen grains, which is crucial for anther opening (Matsui et al., 2000).

Anther dehiscence responds differently to HT stress in maize, rice, and wheat. A long anther dehiscence for pollen release is one trait associated with HT stress tolerance (Matsui and Hasegawa, 2019). Evidence concerning the effects of HT stress on anther dehiscence is limited in wheat, and most studies have revealed that loss of pollen viability is the main factor limiting spikelet fertility under HT stress (Jäger et al., 2008; Prasad and Djanaguiraman, 2014; Begcy et al., 2018). We therefore performed comparisons between maize and rice in the present study (Figure 4E). Rice exhibits apical and bottom dehiscence in the anther, with the latter playing a more crucial role in coping with HT stress (Matsui et al., 2005; Jagadish et al., 2010). A large bottom dehiscence size has been shown to enhance pollen deposition on the stigma (Matsui and Kagata, 2003), and extending the bottom cleft of the anther by 100 μm reduced the incidence of HT-induced sterility by 20% and increased heat tolerance by 0.66°C (Matsui and Hasegawa, 2019). In maize, anther dehiscence occurs only at the apex as a result of deformation of the endothelial wall confined to the tip (Cheng et al., 1979; Keijzer et al., 1996). HT stress during the 14 days bracketing the silking stage had no significant effects on maize anther dehiscence length but significantly reduced anther width, making pollen shedding number the main constraint on seed set (Wang et al., 2020b). At present, there is very limited information on anther apex dehiscence under HT stress in maize.

Pollen germination, tube elongation, and double fertilization

The fertilization process of crops like rice and wheat involves a series of events from pollen capture to gamete fusion (Figure 5; Weterings, 2004; Ge et al., 2007). Upon release from the anthers, pollen grains travel a short distance before being captured by the bifurcated and plumose stigma (phase I). Pollen germination commences within 2–3 min of landing on the stigma, and pollen tubes then penetrate the stigma tissue (phase II) and grow within the style. The tubes continue to grow in the style for 5–10 min (phase III) until entering the various tissue layers of the ovary and reaching the micropyle in about 0.5–1.0 h (phase IV). The male gametes enter the egg cell in approximately 1.5 h, and fusion of female and male nuclei is usually complete at about 5–7 h after pollination (phase V; Figure 5A; Hoshikawa, 1959; Wu and Tsai, 1965; Huang et al., 2004; Chen et al., 2008; Thomas and Franklin-Tong, 2013). By contrast, maize pollen grains have to flow from the tassel (top section of the plant) to the silks (middle section of the plant) and do not begin germinating until 10 min after pollination (Wędzony and Van Lammeren, 1996). Maize pollen tubes develop and grow in a long path (~20 cm) along the transmitting tissues of silks to reach the ovarian cavity (Figure 5C; Miller, 1919), a process that takes 6–24 h, depending upon silk length, much longer than the time required in rice and wheat (Heslop-Harrison et al., 1984; You and Jensen, 1985; Jagadish et al., 2010; Dresselhaus et al., 2011). During this journey, the resources carried in the pollen sustain pollen tube growth for only about 2 cm (Heslop-Harrison et al., 1984); thereafter, the tube becomes increasingly dependent on metabolites from the silks (Dresselhaus et al., 2011). The period from pollen tube arrival at the embryo sac to gamete fusion is relatively short, taking less than 1 h. However, the time from contact to fusion of male and female nucleoli is longer, nearly 5 h in the egg cell and ~3 h in the central cell (Mol et al., 1994). The former then becomes a zygote and develops into a diploid embryo, whereas the latter forms the triploid nutritive endosperm (Lord and Russell, 2002).

The processes of pollen germination, pollen tube growth, and fertilization are all sensitive to HT stress (Dupuis and Dumas, 1990; Kakani et al., 2005; Snider et al., 2011a; Shi et al., 2018; Zhang et al., 2018). When the temperature was over 30°C–35°C, more than 50% of pollen grains failed to germinate in rice (Coast et al., 2016). Similarly, wheat pollen grains germinated *in vitro* under HT stress (34°C/16°C) with a ~40% reduction in pollen germination across genotypes (Bheemanahalli et al., 2019). Pollen viability was significantly reduced at temperatures >38°C in maize (Wang et al., 2021b). Pollen germination failure induced by HT stress is a result of altered pollen morphology (Parish et al., 2012; De Storme and Geelen, 2014; Djanaguiraman et al., 2014), inhibited metabolic processes (Aloni et al., 2001; Karni and Aloni, 2002), and disturbed ROS homeostasis (Rutley et al., 2021; Zhou and Dresselhaus, 2022). It has also been shown that ROS can induce dormancy in some pollen grains under HT stress during the daytime but that the dormant pollen grains germinate at cool temperatures at night, providing new insight for improving heat tolerance during pollination (Rutley et al., 2021). Maize pollen development is particularly sensitive to HT stress, especially at the tetrad stage (Lohani et al., 2020). Temperatures >30°C that occurred at the meiosis phase of wheat pollen development caused pollen abortion (Ullah et al., 2022). A short HT stress at the tetrad stage (35°C/25°C light/dark period for 48 h) reduced pollen germination to ~20% and severely reduced seed set (Begcy et al., 2019; Smith, 2019).

Pollen–stigma interaction, which includes pollen adhesion, hydration, and germination, is critical for successful pollen tube growth from the stigma to the ovule (Dresselhaus and Franklin-Tong, 2013). Under HT stress conditions, pollen–stigma interaction is affected by deformed pollen grains with abnormal pollen wall patterning (especially the exine wall ornamentation; Allen et al.,

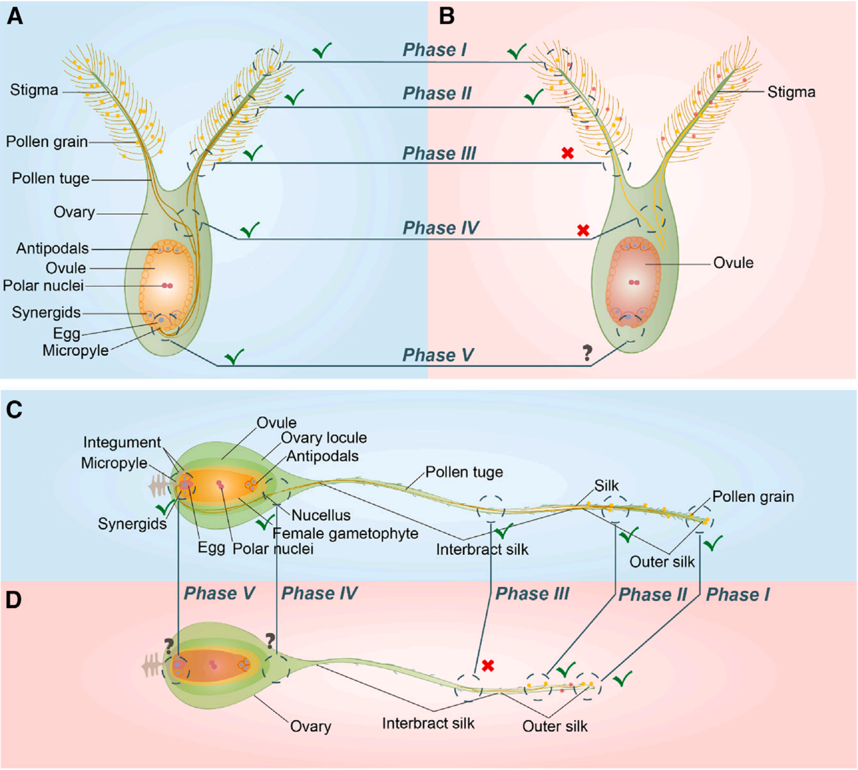

Figure 5. Pollen grain germination and pollen tube elongation on the female reproductive organ (pistil) with a bifurcated plumose stigma in rice and wheat and a filiform stigma in maize under normal temperatures (NT; A and C) and HT stress (B and D).
The graphics illustrate pollen grains shed from the anther (yellow represents pollen grains under normal conditions; red represents pollen grains affected by HT stress) that are captured and adhere to the stigma (phase I). Pollen grains that have germinated on the stigma surface after hydration produce pollen tubes, which penetrate the inner stigma (phase II), grow apically in the pistil transport tract (phase III), pass through the various layers of tissues comprising the ovary (phase IV), and enter the micropyle under the guidance of cell signals (phase V). The pollen tube enters the female gametophyte, interacts with the egg apparatus, and then releases two sperm for double fertilization with the egg cell and polar nuclei, thus initiating early embryo formation and development. Under HT stress, the stigmas on the pistil capture pollen grains, but fewer pollen grains germinate, and pollen tube elongation is hindered. It is difficult for the pollen tube to reach the micropyle for double fertilization. Literature and experimental observations indicate that phases III and/or IV of pollen tube elongation are most sensitive to HT stress. The reddish background represents HT stress, and the blueish background represents normal conditions. The red cross (ч) indicates the HT stress-sensitive phase—a critical factor that affects seed set under HT stress—and the green tick (√) indicates a phase that is relatively tolerant of HT stress. This graphic was drawn based on Thomas and Franklin-Tong (2013), Jagadish (2020), and Lohani et al. (2020), with modification.

2011; Djanaguiraman et al., 2018; Santiago and Sharkey, 2019), as well as low stigmatic receptivity due to increased oxidative stress and reduced soluble carbohydrate and ATP content in the pistil (Snider et al., 2009; Jiang et al., 2019). HT-induced loss of stigmatic receptivity occurs sequentially, first affecting the capacity to assist pollen tube penetration to the transmitting tissue of the pistil, then the ability to support pollen germination, and finally the capacity to maintain pollen grain adhesion (Hedhly et al., 2005). The energy demand of the actively growing pollen tube is nearly 10-fold higher than that of vegetative tissues (Tadege and Kuhlemeier, 1997). HT stress significantly reduced soluble carbohydrate content in the pistil during pollen tube growth and hence reduced pollen tube growth rates in the transmitting tissue (Snider et al., 2011a, 2011b). Auxin, flavonol, and ROS homeostasis in the pistils also play key roles in pollen germination and tube elongation in rice (Muhlemann et al., 2018; Zhang et al., 2018). Reduced auxin levels in the pistils of heat-sensitive rice genotypes slow pollen tube growth under HT stress (Zhang et al., 2018). Flavonol maintains the integrity of pollen tube growth by regulating the dynamic balance of ROS (Muhlemann et al., 2018). In wheat, heat-stressed stigmas had similar numbers of germinated pollen grains compared with non-stressed stigmas, but fewer pollen tubes reached the ovary (Saini et al., 1983). Damaged female organs (stigma and style) cannot provide clear guidance that typically comes from synergid cells in the embryo sac after pollen germination, resulting in undirected pollen tube growth (Higashiyama et al., 2001; Okuda et al., 2009; Snider et al., 2009). These previous results were also confirmed by our recent studies in maize. Compared with pollen adherence and germination on the stigma, pollen tube growth in the transmitting tissue of the style/silk and ovary (phase III and/or IV) is expected to be more sensitive to HT stress (Figures 5B and 5D), closely associated with reduced energy supply from the silk and increased ABA and ROS content (Rezaul et al., 2019). In addition, degenerated eggs and synergids, malformed embryo sacs, and more aberrant or abortive ovules have been observed in heat-stressed pistils of tomato, canola (*Brassica napus* L.), wheat, and cotton (*Gossypium hirsutum* L.) (Iwahori, 1965; Saini et al., 1983; Polowick and Sawhney, 1988; Snider et al., 2009; Djanaguiraman et al., 2018). These can result in fertilization failure, directly or indirectly, by hindering pollen tube burst and sperm cell release (Dresselhaus and Franklin-Tong, 2013).

The findings above suggest that the hidden stigmas of monoecious crops such as rice and wheat are beneficial for resisting or escaping from HT stress compared with exserted anthers (Supplemental Figure 3). But for maize, the silks must expose themselves to hot, dry air to receive pollen grains under HT stress conditions, and this is expected to reduce stigma receptivity and pistil energy supply. Pollen shedding, pollen germination, and initial growth of the pollen tube also involve exposure to the outside environment, increasing the difficulty of successful maize pollination under HT stress. Moreover, the long maize silk requires a long period of pollen tube growth, further increasing the HT stress sensitivity of seed set in maize. Nonetheless, little detailed information is available on stigma

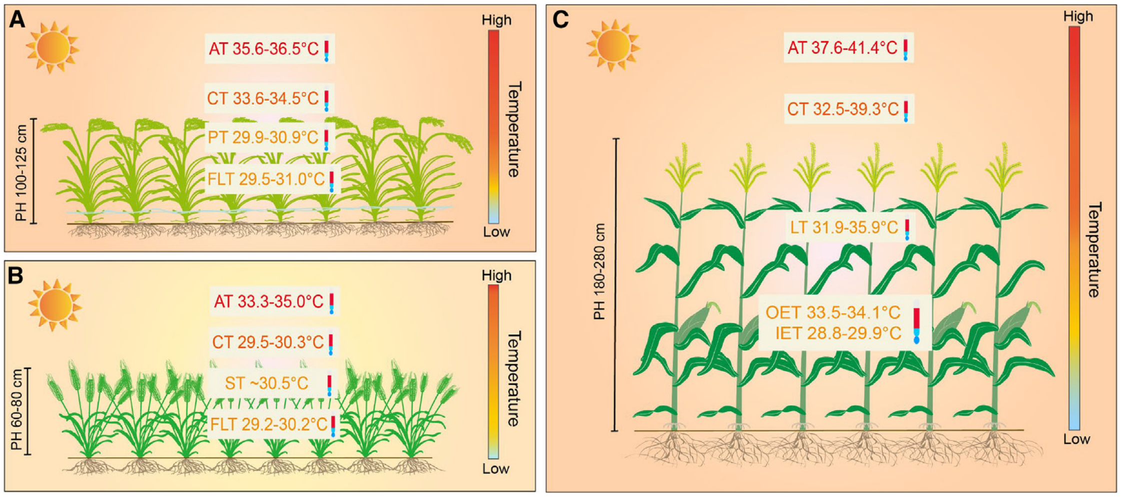

Figure 6. Air, canopy, and organ (flag leaf, panicle/spike/ear) temperatures of three main global cereal crop populations during flowering under field conditions.

Because of variations in sowing date and growing environment, rice and maize are more likely to experience HT stress. The reddish background for rice and maize represents more severe HT stress, and the yellowish background for wheat represents low HT stress. The scales at the right side of each crop population represent the change in temperature from the ground to 2 m above the ground. The range of temperature change varies with the vertical height of different crop populations (e.g., plant height) and growing environments (e.g., flooded vs. dry land).

(A) Temperature levels of different heights of rice populations were obtained from field experiments performed at the Jiangning Experimental Station (118°30′E, 31°50′N), Jiangsu Province, China (see Yan et al., 2010). Temperature was measured at the heading and anthesis stages on September 11 and August 25, 2017.

(B) Temperature data for the wheat population were obtained during the 2000–2001 winter–spring growing season at CIMMYT's experimental station, Ciudad Obregon (109°56′W, 27°29′N), Mexico (see Ayeneh et al., 2002).

(C) Temperature data for the maize population were obtained from a field experiment at the ICAR-Central Research Institute for Dryland Agriculture (78°31′E, 17°21′N), Santoshnagar, Hyderabad, India, during the 2013 summer season (see Yadav et al., 2015). The outer and inner ear temperature data were obtained from a field experiment performed at the Wuqiao Experiment station (116°30′E, 37°36′N; Wang et al., 2023), Hebei Province, China. The outer and inner ear temperatures were recorded at 10-s intervals for 12 h using a microthermometer with a microneedle thermometer sensor and a microlinear thermometer sensor. Temperature was measured from 13:00 to 14:00. AT, air temperature; CT, canopy temperature; PT, panicle temperature; ST, spike temperature; FLT, flag leaf temperature; LT, leaf temperature; OET, outer ear temperature; IET, inner ear temperature; PH, plant height.

receptivity, pollen tube growth, and fertilization in cereal crops, especially maize .

Canopy and tissue temperatures upon HT stress during flowering

Studies of the effects of HT stress on crop seed set generally rely on temperature-controlled experiments or field experiments over large regions in which temperatures are measured by standard weather stations at a 2-m height (Jagadish et al., 2007; Siebert et al., 2014; Aiqing et al., 2018; Wang et al., 2019b; Liu et al., 2022a). In natural field conditions, yield reduction in wheat as a result of HT stress during flowering is underestimated when temperature is measured at a 2-m height (Siebert et al., 2014), highlighting the importance of canopy temperature for reducing the uncertainty in assessing HT stress effects on crop yield (Ayeneh et al., 2002). Temperatures of leaf, spike, and panicle tissues are lower than air temperatures and are more significantly correlated with spikelet sterility during flowering in rice (Rajendran et al., 2016). Lower panicle temperature is expected to reduce the effects of HT stress during flowering on seed set (Mackill and Coffman, 1983) and can partly explain the relatively high panicle fertility of rice subjected to HTs >35°C at the heading and flowering stages (Julia and Dingkuhn, 2013). The characteristics of these plant tissues have important implications for selecting and breeding HT-tolerant crop varieties. The present results based on previously published field work showed that canopy temperature (CT) was higher than panicle/spike/ear temperature in rice, wheat, and maize but lower than air temperature (AT) above the canopy (Figure 6), consistent with previous findings (Ayeneh et al., 2002; Yan et al., 2010; Yadav et al., 2015). Lower canopy and tissue temperatures result from the strong interaction between AT and relative humidity (RH; Matsui et al., 1997; Yan et al., 2010). In conditions with low RH, transpiration increases, which lowers the temperature of tissues, particularly leaves (Rajendran et al., 2016; Lin et al., 2017; Drake et al., 2018). During a heat wave, transpiration provides substantial latent cooling (an average cooling of ~2.8°C; Drake et al., 2018), which serves as an important response to HT stress. Other factors, such as soil moisture, canopy roughness, plant height, and leaf physical traits, also affect the extent of AT reduction (Fuchs, 1990; Stockle and Dugas, 1992; Julia and Dingkuhn, 2013; Lin et al., 2017). In the case of rice grown in paddy fields, the presence of a ponded water layer in the irrigation system can change the microclimate within the canopy population (Stuerz et al., 2014). Under these conditions, the flag leaf temperature can be ~5°C lower than AT because of the cooling effect of the buffering layer of ponded water (Figure 6A; Yan et al., 2010). The soil temperature is also lowered, and root activity can therefore

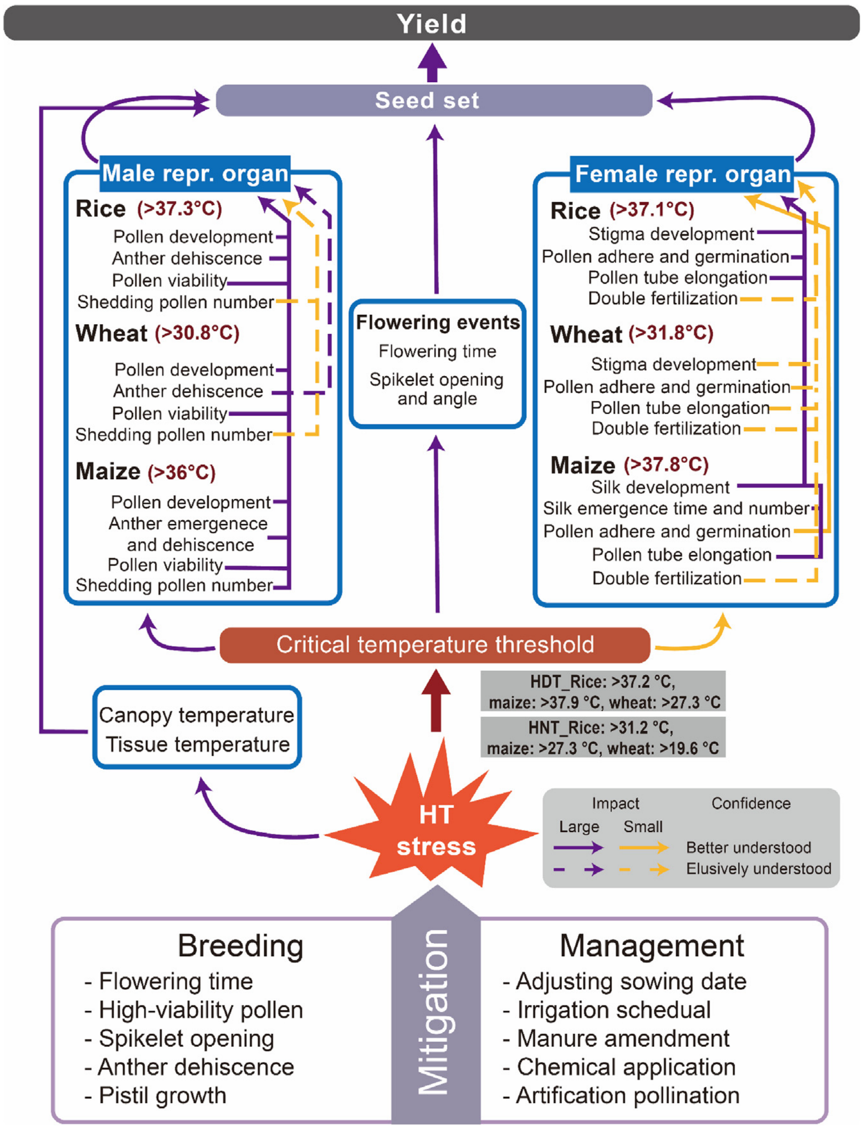

Figure 7. A wiring diagram for the response of male and female reproductive organs to HT stress, critical temperature thresholds, and canopy (tissue) temperatures in rice, wheat, and maize during the flowering stage.

A comprehensive list of selection indexes for breeding HT-tolerant varieties and crop production management strategies for coping with HT stress is provided. The color of the wire indicates the expected magnitude of the effect on seed set and yield formation, and the shape of the wire reflects the level of knowledge underpinning the link represented by the wire.

be maintained at a high level under HT conditions after application of saturated irrigation at the post-heading stage (Matsue et al., 2021). In some rice varieties, the cooling effects of the water layer on canopy, tissues, and organs have enabled spikelet number and fertility to be maintained even in controlled environments up to 39°C (Jagadish et al., 2015).

In wheat, the growing environment does not typically expose plants to HT stress of >38°C during flowering. Under natural field conditions, ATs experienced by wheat can range from 33.3°C to 35°C, and the CT, spike temperature, and flag leaf temperature are lower: 29.5°C to 30.3°C, ~30.5°C, and 29.2°C to 30.2°C, respectively (Figure 6B; Ayeneh et al., 2002). Temperature depression in the canopy and tissues was mainly attributed to transpirational cooling mediated by low RH (Yan et al., 2010; Lin et al., 2017). In irrigated agriculture, HT stress in wheat is considerably reduced owing to surface cooling (Siebert et al., 2017).

In maize, tall plants (~2 m in height) cause the top of the canopy to be close to the height at which weather stations measure AT, but a significant reduction in temperature still exists between AT, CT, and leaf temperature (LT) (Yadav et al., 2015). During anthesis, when ATs ranged from 37.6°C to 41.4°C, CT and leaf temperature ranged from 32.5°C–39.3°C and 31.9°C–35.9°C, respectively (Figure 6C). Because of the different locations of male and female reproductive organs on maize plants, the tassel has a higher tissue temperature, and the ear located at the middle section of the plant has a lower tissue temperature. Moreover, ear temperature declines significantly from the outer to the inner side because of the thick husk leaves, which can shield pollen tube growth and fertilization processes from HT stress (Wang et al., 2023). When the AT was as high as 40°C, the outer ear temperature ranged from 33.5°C to 34.1°C, whereas the inner ear temperature ranged from 28.8°C to 29.9°C (Figure 6C).

Above all, accurate assessment of the effects of HT stress requires a precise understanding of the responses of canopy and tissue temperatures (especially reproductive tissues) to AT. CT is widely recognized as a reliable indicator for assessing crop yield losses under HT stress (Siebert et al., 2014, 2017; Webber et al., 2016). Lowering reproductive tissue temperatures is beneficial for maintaining stable spikelet fertility under HT stress (Yan et al., 2010; Bonari et al., 2020). Currently, we have limited information on the critical thresholds of canopy/tissue temperatures for seed set at different reproductive growth stages in the three staple crops.

Concluding remarks and future perspectives

As the climate warms, crop grain yield is at risk from increasing frequency, duration, and intensity of HT stress. Hence, it is becoming especially important to ensure high seed set/spikelet fertility under HT stress. In this study, we used the concept of a wiring diagram to summarize the impact level and the current state of knowledge about key reproductive events that affect seed set in rice, wheat, and maize (Figure 7). When

the temperature exceeds the critical temperature thresholds, crop growth and development and the processes of seed formation become "derailed," resulting in a significant reduction in seed number (Sanchez et al., 2014; Wang et al., 2021b; Zhu et al., 2021). Until recently, the effects of HDT during flowering on crop reproductive events have been more clearly understood than the effects of HNT. Because of the limited research on HNT, we tentatively derived critical HNT thresholds for seed set in rice (31.2°C ± 0.2°C), maize (27.3°C ± 1.3°C), and wheat (19.6°C ± 2.7°C). The mechanisms underlying the effects of HNT during flowering on seed set are relatively poorly understood, as the phenomenon of anomalous nighttime warming has not been closely examined until recent years. More research should be devoted to revealing the negative effects of HNT on crop fertility at the phenotypic, physiological, and molecular levels.

Crops have different HT stress sensitivities at different reproductive phases, corresponding to different critical air, canopy, and tissue temperature thresholds for growth, development, and seed set (Figures 2, 3, and 6). Rice, wheat, and maize are extremely sensitive to HT stress during the early stage of reproductive development (i.e., the microspore stage) and at the pollination stage (Yoshida et al., 1981; Endo et al., 2009; Wassmann et al., 2009; Wang et al., 2020a). Asynchronies between individual male and female flowers in growth, development, and flowering increase the complexity of the HT stress response in maize (Wang et al., 2020a). Heat damage to pollen development, anther dehiscence, and pollen viability greatly reduces the fertility of male reproductive organs (Matsui et al., 2000; Endo et al., 2009; Begcy et al., 2019; Matsui and Hasegawa, 2019; Hu et al., 2021). The effect of pollen shedding number on seed set has rarely been examined in rice and wheat under HT stress owing to the difficulty of quantifying pollen grains. Presumably, pollen shedding number is not a key factor affecting seed set in these two crops, as they exhibit cleistogamy and autogamy (Supplemental Figures 3A, 3B, 3D, and 3E). Compared with that in rice and wheat, the fertility of male reproductive organs in maize is more sensitive to heat damage. Spikelet opening is a pre-condition for pollination in maize, and pollen release from the anther is expected to be more difficult in maize than in rice (Figure 4E). At present, there are fewer studies on HT stress in female reproductive organs of the three crops. HT effects on stigma exposure, pollen adherence and germination on the stigma, pollen tube growth, and fertilization have been detailedly studied in rice. In maize, HT stress disrupts silk development (advancing or delaying silk emergence, depending on the HT level), reduces silk emergence number, and limits silk viability. Surprisingly, pollen tube growth in the pistil tissue transmitting tract is more sensitive than pollen germination to HT stress, especially in maize (Figure 5). The occurrence of a heat spell at noon frequently coincides with pollen tube growth and reduces spikelet fertility, an important but often neglected phenomenon in research and practice.

How to reduce the effects of HT stress on crop yield is becoming an increasingly urgent question, and the answer will depend mainly on improvements in crop breeding and management. The present study reveals several parameters that can be used as selection indexes in breeding and selection for HT tolerance and avoidance during flowering in the three staple crops (Figure 7). Some important selection indexes and their regulatory mechanisms are listed below.

(1) Flowering time in days (blooming synchronicity between male and female flowers) and hours (early-morning flowering to escape heat damage at noon).
  - A quantitative trait locus (*qEMF3*) in rice shifts flower opening time 1.5–2 h earlier so that flower opening is completed before the temperature reaches 35°C in the late morning, thus mitigating heat-induced spikelet sterility under diverse environmental conditions (Hirabayashi et al., 2015; Ishimaru et al., 2022).

(2) Pollen production in the anther, pollen shedding number, and pollen viability to maintain high spikelet fertility at different reproductive phases.
  - *OsmiRNA528* improves pollen development in rice by inducing *OsUCL23* gene expression to influence flavonoid metabolism (Zhang et al., 2020).

(3) Spikelet opening angle, lodicule size, and lodicule structure to ensure anther emergence under HT stress, especially in maize.
  - *Diurnal Flower Opening Time 1* (*DFOT1*) modulates pectin methylesterase activity to regulate pectin methylesterification levels of lodicule cell walls, thereby affecting lodicule swelling and spikelet opening time in rice (Wang et al., 2022).
  - *Sterol methyltransferase2* controls lodicule swelling in maize and thus affects spikelet opening and anther emergence (Liu et al., 2023).

(4) Anther dehiscence to enhance pollen release (length of bottom dehiscence is more important for rice, and length of apical dehiscence is critical for maize).
  - Basal dehiscence length (*qBDL2-2* and *qBDL10*) has a significant effect on heat tolerance in rice and can be used in heat-tolerance breeding (Zhao et al., 2016).

(5) Pistil growth and viability to receive pollen grains and protect pollen germination and pollen tube growth in the transmitting tract (silk elongation, uniformity of silk emergence at different cob positions, and silk receptivity are particularly important factors that affect seed set under HT stress in maize).
  - Loss of *KIRA1-LIKE1* function extends the duration of silk receptivity and thus markedly increases kernel set (Šimášková et al., 2022).

Existing varieties of the three crops still exhibit limited HT stress tolerance, especially under extreme heat waves. Crop management is likely to have expected effects on reducing damage due to HT stress (Figure 7). Based on the present study, suitable crop management practices that can reduce and/or avoid HT stress damage during flowering should include the following.

(1) Adjustment of sowing date to avoid HT events coinciding with HT-sensitive stages of sexual reproduction (e.g., anthesis; sowing date is generally advanced to avoid HT stress at anthesis).
  - Adjusting the sowing date of maize increased kernel number per ear in contrasting genetic backgrounds by 70% by avoiding heat stress at the flowering stage under natural field conditions (Liu et al., 2022b).

(2) Irrigation to reduce soil, canopy, and tissue temperatures and maintain the structure and function of plant organs

by supplying water; it can be applied as a preventive or mitigation measure shortly before or after occurrences of HT stress.

- Irrigation scheduling can effectively reduce CT, thereby mitigating reductions in grain yield of maize under HT stress (Majumder et al., 2016).
- After heading time, application of saturated irrigation in a rice paddy can lower the soil temperature, maintain root activity, and thus improve rice yield (Matsue et al., 2021).

(3) Manure amendment to improve soil fertility and soil moisture content, a preventive management strategy that can be performed at sowing or well before HT stress.

- Manure amendment could potentially reduce global yield losses induced by extreme HT from 33.6% to 25.1% in rice (Zhu et al., 2022).

(4) Chemical spraying to enhance the function of specific plant organs, a specific management practice performed immediately before and after HT stress.

- Application of JA and BRs can enhance spikelet opening of rice and maize by promoting lodicule swelling (Yang et al., 2020; Liu et al., 2023).
- Chemical application (with 24-epibrassinolide as the main component) effectively enhanced spikelet fertility by 60.5% in heat-sensitive rice by increasing pollen germination and promoting pollen tube growth, thereby alleviating HT stress injury during fertilization (Wu et al., 2020).

(5) Artificial pollination (with drones or bamboo poles) to promote pollen shedding and increase the number of pollen grains on the stigma before occurrences of HT stress, a flexible management strategy for coping with HT stress on daily and hourly timescales.

- Intensified pollination (with bamboo poles) effectively enhanced spikelet fertility of heat-sensitive rice by 27.4% during flowering (Wu et al., 2020).
- Drone-assisted pollination improves pollination efficiency under HT stress during flowering (Rehna and Inamdar, 2022; Weng et al., 2022).

DATA AVAILABILITY

The data that support the findings of this study are available upon reasonable request from the corresponding author.

Data on the monthly average maximum temperatures of rice, wheat, and maize in different stations of each country were obtained from https://www.worldclim.org/data/monthlywth.html.

Data on the global average near-surface temperature anomaly relative to 1961–1990 were obtained from https://hadleyserver.metoffice.gov.uk/hadcrut5.

Data on the critical temperatures for key flowering traits of rice, wheat, and maize around the flowering stage (Figure 3) were extracted from the references listed in the supplemental information.

SUPPLEMENTAL INFORMATION

Supplemental information is available at *Plant Communications Online*.

FUNDING

This research was supported by the National Science Foundation of China (32272214), the 2115 Talent Development Program of China Agricultural University, and the General Project of Chongqing Natural Science Foundation (cstc2021jcyj-msxmX0747).

AUTHOR CONTRIBUTIONS

M.L. and S.H. planned and designed the topic. M.L. wrote the manuscript. Y.Z., J.S., Q.Y., B.L., and Y.W. helped with figures and data collection. S.H., F.M., Y.G., X.D., S.L., and P.W. supervised and helped with manuscript revision.

ACKNOWLEDGMENTS

No conflict of interest is declared.

Received: February 20, 2023
Revised: April 29, 2023
Accepted: May 22, 2023
Published: May 23, 2023

REFERENCES

Abiko, M., Akibayashi, K., Sakata, T., Kimura, M., Kihara, M., Itoh, K., Asamizu, E., Sato, S., Takahashi, H., and Higashitani, A. (2005). High-temperature induction of male sterility during barley (*Hordeum vulgare* L.) anther development is mediated by transcriptional inhibition. Sex. Plant Reprod. **18**:91–100.

Aiqing, S., Somayanda, I., Sebastian, S.V., Singh, K., Gill, K., Prasad, P.V.V., and Jagadish, S.V.K. (2018). Heat stress during flowering affects time of day of flowering, seed set, and grain quality in spring wheat. Crop Sci. **58**:380–392.

Alberto, M.C.R., Wassmann, R., Hirano, T., Miyata, A., Kumar, A., Padre, A., and Amante, M. (2009). CO_2/heat fluxes in rice fields: comparative assessment of flooded and non-flooded fields in the Philippines. Agric. For. Meteorol. **149**:1737–1750.

Allen, A.M., Thorogood, C.J., Hegarty, M.J., Lexer, C., and Hiscock, S.J. (2011). Pollen–pistil interactions and self-incompatibility in the Asteraceae: new insights from studies of Senecio squalidus. Ann. Bot. **108**:687–698.

Aloni, B., Peet, M., Pharr, M., and Karni, L. (2001). The effect of high temperature and high atmospheric CO2 on carbohydrate changes in bell pepper (*Capsicum annuum*) pollen in relation to its germination. Physiol. Plantarum **112**:505–512.

Arshad, M.S., Farooq, M., Asch, F., Krishna, J.S.V., Prasad, P.V.V., and Siddique, K.H.M. (2017). Thermal stress impacts reproductive development and grain yield in rice. Plant Physiol. Biochem. **115**:57–72.

Ayeneh, A., Van Ginkel, M., Reynolds, M., and Ammar, K. (2002). Comparison of leaf, spike, peduncle and canopy temperature depression in wheat under heat stress. Field Crop. Res. **79**:173–184.

Barber, H.M., Lukac, M., Simmonds, J., Semenov, M.A., and Gooding, M.J. (2017). Temporally and genetically discrete periods of wheat sensitivity to high temperature. Front. Plant Sci. **8**:51.

Beauzamy, L., Nakayama, N., and Boudaoud, A. (2014). Flowers under pressure: ins and outs of turgor regulation in development. Ann. Bot. **114**:1517–1533.

Begcy, K., Nosenko, T., Zhou, L.Z., Fragner, L., Weckwerth, W., and Dresselhaus, T. (2019). Male sterility in maize after transient heat stress during the tetrad stage of pollen development. Plant Physiol. **181**:683–700.

Begcy, K., Weigert, A., Egesa, A., and Dresselhaus, T. (2018). Compared to Australian cultivars, European summer wheat (*Triticum aestivum*) overreacts when moderate heat stress is applied at the pollen development stage. Agronomy **8**:99.

Bheemanahalli, R., Sunoj, V.S.J., Saripalli, G., Prasad, P.V.V., Balyan, H.S., Gupta, P.K., Grant, N., Gill, K.S., and Jagadish, S.V.K. (2019). Quantifying the impact of heat stress on pollen germination, seed set, and grain filling in spring wheat. Crop Sci. **59**:684–696.

Bheemanahalli, R., Impa, S.M., Krassovskaya, I., Vennapusa, A.R., Gill, K.S., Obata, T., and Jagadish, S.V.K. (2020). Enhanced

N-metabolites, ABA and IAA-conjugate in anthers instigate heat sensitivity in spring wheat. Physiol. Plantarum **169**:501–514.

Bonari, A., Edalat, M., Ghadiri, H., Kazemeini, S.A., and Modarresi, M. (2020). The study of temperature depression and its association with grain yield in six wheat cultivars under heat stress conditions and salicylic acid application. Iran Agric. Res. **39**:99–108.

Borrás, L., Westgate, M.E., Astini, J.P., and Echarte, L. (2007). Coupling time to silking with plant growth rate in maize. Field Crop. Res. **102**:73–85.

Bortiri, E., and Hake, S. (2007). Flowering and determinacy in maize. J. Exp. Bot. **58**:909–916.

Cai, Q., Yuan, Z., Chen, M., Yin, C., Luo, Z., Zhao, X., Liang, W., Hu, J., and Zhang, D. (2014). Jasmonic acid regulates spikelet development in rice. Nat. Commun. **5**:3476.

Cantarero, M.G., Cirilo, A.G., and Andrade, F.H. (1999). Night temperature at silking affects kernel set in maize. Crop Sci. **39**:703–710.

Cárcova, J., Andrieu, B., and Otegui, M.E. (2003). Silk elongation in maize: relationship with flower development and pollination. Crop Sci. **43**:914–920.

Cecchetti, V., Altamura, M.M., Falasca, G., Costantino, P., and Cardarelli, M. (2008). Auxin regulates arabidopsis anther dehiscence, pollen maturation, and filament elongation. Plant Cell **20**:1760–1774.

Chakraborty, D., Sehgal, V.K., Dhakar, R., Ray, M., and Das, D.K. (2019). Spatio-temporal trend in heat waves over India and its impact assessment on wheat crop. ThApC **138**:1925–1937.

Chen, J., Xu, Y., Fei, K., Wang, R., He, J., Fu, L., Shao, S., Li, K., Zhu, K., Zhang, W., et al. (2020). Physiological mechanism underlying the effect of high temperature during anthesis on spikelet-opening of photo-thermo-sensitive genic male sterile rice lines. Sci. Rep. **10**:2210.

Chen, S.-q., Zhong, W., Liu, M.-x., Xie, Z.-w., and Wang, H.-h. (2008). Pollen grain germination and pollen tube growth in pistil of rice. Rice Sci. **15**:125–130.

Chen, Y., Ma, J., Miller, A.J., Luo, B., Wang, M., Zhu, Z., and Ouwerkerk, P.B.F. (2016). OsCHX14 is involved in the K+ homeostasis in rice (*Oryza sativa*) flowers. Plant Cell Physiol. **57**:1530–1543.

Cheng, P.C., Greyson, R.I., and Walden, D.B. (1979). Comparison of anther development in genic male-sterile (ms10) and in male-fertile corn (*Zea mays*) from light microscopy and scanning. Can. J. Bot. **57**:578–596.

Cheng, W., Sakai, H., Yagi, K., and Hasegawa, T. (2009). Interactions of elevated [CO_2] and night temperature on rice growth and yield. Agric. For. Meteorol. **149**:51–58.

Cicchino, M., Edreira, J.I.R., and Otegui, M. (2010). Heat stress during late vegetative growth of maize: effects on phenology and assessment of optimum temperature. Crop Sci. **50**:1431–1437.

Coast, O., Ellis, R.H., Murdoch, A.J., Quiñones, C., and Jagadish, K.S.V. (2015). High night temperature induces contrasting responses for spikelet fertility, spikelet tissue temperature, flowering characteristics and grain quality in rice. Funct. Plant Biol. **42**:149–161.

Coast, O., Murdoch, A.J., Ellis, R.H., Hay, F.R., and Jagadish, K.S.V. (2016). Resilience of rice (*Oryza spp*.) pollen germination and tube growth to temperature stress. Plant Cell Environ. **39**:26–37.

Cui, Z., Dong, H., Zhang, A., Ruan, Y., He, Y., and Zhang, Z. (2020). Assessment of the potential for genomic selection to improve husk traits in maize. G3 **10**:3741–3749.

De Jong, T., and Klinkhamer, P. (2005). Evolutionary Ecology of Plant Reproductive Strategies (Cambridge University Press).

De Storme, N., and Geelen, D. (2014). The impact of environmental stress on male reproductive development in plants: biological processes and molecular mechanisms. Plant Cell Environ. **37**:1–18.

De Vries, A.P. (1971). Flowering biology of wheat, particularly in view of hybrid seed production—a review. Euphytica **20**:152–170.

Dellaporta, S.L., and Calderon-Urrea, A. (1994). The sex determination process in maize. Science **266**:1501–1505.

Denisow, B., Masierowska, M., Winiarczyk, K., and Rakoczy-Trojanowska, M. (2022). The pollen dispersal ability for cross-pollination in winter wheat (*Triticum aestivum* L.) is related to anther extrusion capability rather than to pollen output. South Afr. J. Bot. **148**:283–292.

Djanaguiraman, M., Narayanan, S., Erdayani, E., and Prasad, P.V.V. (2020). Effects of high temperature stress during anthesis and grain filling periods on photosynthesis, lipids and grain yield in wheat. BMC Plant Biol. **20**:268.

Djanaguiraman, M., Perumal, R., Jagadish, S.V.K., Ciampitti, I.A., Welti, R., and Prasad, P.V.V. (2018). Sensitivity of sorghum pollen and pistil to high-temperature stress. Plant Cell Environ. **41**:1065–1082.

Djanaguiraman, M., Vara Prasad, P.V., Murugan, M., Perumal, R., and Reddy, U.K. (2014). Physiological differences among sorghum (*Sorghum bicolor* L. *Moench*) genotypes under high temperature stress. Environ. Exp. Bot. **100**:43–54.

Dong, X., Guan, L., Zhang, P., Liu, X., Li, S., Fu, Z., Tang, L., Qi, Z., Qiu, Z., Jin, C., et al. (2021). Responses of maize with different growth periods to heat stress around flowering and early grain filling. Agric. For. Meteorol. **303**:108378.

Drake, J.E., Tjoelker, M.G., Vårhammar, A., Medlyn, B.E., Reich, P.B., Leigh, A., Pfautsch, S., Blackman, C.J., López, R., Aspinwall, M.J., et al. (2018). Trees tolerate an extreme heatwave via sustained transpirational cooling and increased leaf thermal tolerance. Global Change Biol. **24**:2390–2402.

Dresselhaus, T., and Franklin-Tong, N. (2013). Male–female crosstalk during pollen germination, tube growth and guidance, and double fertilization. Mol. Plant **6**:1018–1036.

Dresselhaus, T., Lausser, A., and Márton, M.L. (2011). Using maize as a model to study pollen tube growth and guidance, cross-incompatibility and sperm delivery in grasses. Ann. Bot. **108**:727–737.

Dupuis, I., and Dumas, C. (1990). Influence of temperature stress on in vitro fertilization and heat shock protein synthesis in maize (*Zea mays* L.) reproductive tissues. Plant Physiol. **94**:665–670.

Rattalino Edreira, J., Budakli Carpici, E., Sammarro, D., and Otegui, M.E. (2011). Heat stress effects around flowering on kernel set of temperate and tropical maize hybrids. Field Crop. Res. **123**:62–73.

Endo, M., Tsuchiya, T., Hamada, K., Kawamura, S., Yano, K., Ohshima, M., Higashitani, A., Watanabe, M., and Kawagishi-Kobayashi, M. (2009). High temperatures cause male sterility in rice plants with transcriptional alterations during pollen development. Plant Cell Physiol. **50**:1911–1922.

Fábián, A., Sáfrán, E., Szabó-Eitel, G., Barnabás, B., and Jäger, K. (2019). Stigma functionality and fertility are reduced by heat and drought co-stress in wheat. Front. Plant Sci. **10**:244.

Fu, G., Feng, B., Zhang, C., Yang, Y., Yang, X., Chen, T., Zhao, X., Zhang, X., Jin, Q., and Tao, L. (2016). Heat stress is more damaging to superior spikelets than inferiors of rice (*Oryza sativa* L.) due to their different organ temperatures. Front. Plant Sci. **7**:1637.

Fuchs, M. (1990). Infrared measurement of canopy temperature and detection of plant water stress. Theor. Appl. Climatol. **42**:253–261.

Fujioka, S., and Yokota, T. (2003). Biosynthesis and metabolism of brassinosteroids. Annu. Rev. Plant Biol. **54**:137–164.

Gao, J., Wang, S., Zhou, Z., Wang, S., Dong, C., Mu, C., Song, Y., Ma, P., Li, C., Wang, Z., et al. (2019). Linkage mapping and genome-wide association reveal candidate genes conferring thermotolerance of seed-set in maize. J. Exp. Bot. **70**:4849–4864.

García, G.A., Dreccer, M.F., Miralles, D.J., and Serrago, R.A. (2015). High night temperatures during grain number determination reduce wheat and barley grain yield: a field study. Global Change Biol. **21**:4153–4164.

Ge, L.L., Tian, H.Q., and Russell, S.D. (2007). Calcium function and distribution during fertilization in angiosperms. Am. J. Bot. **94**:1046–1060.

Giorno, F., Wolters-Arts, M., Mariani, C., and Rieu, I. (2013). Ensuring reproduction at high temperatures: the heat stress response during anther and pollen development. Plants **2**:489–506.

Gol, L., Tomé, F., and von Korff, M. (2017). Floral transitions in wheat and barley: interactions between photoperiod, abiotic stresses, and nutrient status. J. Exp. Bot. **68**:1399–1410.

Gourdji, S.M., Sibley, A.M., and Lobell, D.B. (2013). Global crop exposure to critical high temperatures in the reproductive period: historical trends and future projections. Environ. Res. Lett. **8**:024041.

Grossiord, C., Buckley, T.N., Cernusak, L.A., Novick, K.A., Poulter, B., Siegwolf, R.T.W., Sperry, J.S., and McDowell, N.G. (2020). Plant responses to rising vapor pressure deficit. New Phytol. **226**:1550–1566.

Hassan, M.U., Chattha, M.U., Khan, I., Chattha, M.B., Barbanti, L., Aamer, M., Iqbal, M.M., Nawaz, M., Mahmood, A., Ali, A., et al. (2020). Heat stress in cultivated plants: nature, impact, mechanisms, and mitigation strategies—a review. Plant Biosystems - An International Journal Dealing with all Aspects of Plant Biology **155**:211–234.

Hays, D.B., Do, J.H., Mason, R.E., Morgan, G., and Finlayson, S.A. (2007). Heat stress induced ethylene production in developing wheat grains induces kernel abortion and increased maturation in a susceptible cultivar. Plant Sci. **172**:1113–1123.

Hedhly, A., Hormaza, J.I., and Herrero, M. (2005). The effect of temperature on pollen germination, pollen tube growth, and stigmatic receptivity in peach. Plant Biol. **7**:476–483.

Hedhly, A., Hormaza, J.I., and Herrero, M. (2009). Global warming and sexual plant reproduction. Trends Plant Sci. **14**:30–36.

Heslop-Harrison, Y., and Heslop-Harrison, J.S. (1996). Lodicule function and filament extension in the grasses: potassium ion movement and tissue specialization. Ann. Bot. **77**:573–582.

Heslop-Harrison, Y., Reger, B.J., and Heslop-Harrison, J. (1984). The pollen-stigma interaction in the grasses. 6. The stigma ('silk') of *Zea mays* L. as host to the pollens of *Sorghum bicolor* (L.) *Moench* and *Pennisetum americanum* (L.) Leeke. Acta Bot. Neerl. **33**:205–227.

Hirabayashi, H., Sasaki, K., Kambe, T., Gannaban, R.B., Miras, M.A., Mendioro, M.S., Simon, E.V., Lumanglas, P.D., Fujita, D., Takemoto-Kuno, Y., et al. (2015). qEMF3, a novel QTL for the early-morning flowering trait from wild rice, Oryza officinalis, to mitigate heat stress damage at flowering in rice. J. Exp. Bot. **66**:1227–1236.

Higashiyama, T., Yabe, S., Sasaki, N., Nishimura, Y., Miyagishima, S.-y., Kuroiwa, H., and Kuroiwa, T. (2001). Pollen tube attraction by the synergid cell. Science **293**:1480–1483.

Honda, I., Turuspekov, Y., Komatsuda, T., and Watanabe, Y. (2005). Morphological and physiological analysis of cleistogamy in barley. Hordeum vulgare **124**:524–531.

Hoshikawa, K. (1959). Cytological studies of double fertilization in wheat (*Triticum aestivum* L.). Jpn. J. Crop Sci. **28**:142–146.

Hu, J., Mitchum, M.G., Barnaby, N., Ayele, B.T., Ogawa, M., Nam, E., Lai, W.-C., Hanada, A., Alonso, J.M., Ecker, J.R., et al. (2008). Potential sites of bioactive gibberellin production during reproductive growth in arabidopsis. Plant Cell **20**:320–336.

Hu, Q., Wang, W., Lu, Q., Huang, J., Peng, S., and Cui, K. (2021). Abnormal anther development leads to lower spikelet fertility in rice (*Oryza sativa* L.) under high temperature during the panicle initiation stage. BMC Plant Biol. **21**:428.

Huang, J.H., Wang, G.J., Sun, Y., and Diao, Y.L. (2004). Studies on the formation date of pollen tube way of wheat. Heilongjiang Agri Sci **20-22**.

Impa, S.M., Raju, B., Hein, N.T., Sandhu, J., Prasad, P.V.V., Walia, H., and Jagadish, S.V.K. (2021). High night temperature effects on wheat and rice: current status and way forward. Plant Cell Environ. **44**:2049–2065.

Ishiguro, S., Kawai-Oda, A., Ueda, J., Nishida, I., and Okada, K. (2001). The *defective in anther DEHISCENCE1* gene encodes a novel phospholipase A1 catalyzing the initial step of jasmonic acid biosynthesis, which synchronizes pollen maturation, anther dehiscence, and flower opening in arabidopsis. Plant Cell **13**:2191–2209.

Ishimaru, T., Hirabayashi, H., Ida, M., Takai, T., San-Oh, Y.A., Yoshinaga, S., Ando, I., Ogawa, T., and Kondo, M. (2010). A genetic resource for early-morning flowering trait of wild rice Oryza officinalis to mitigate high temperature-induced spikelet sterility at anthesis. Ann. Bot. **106**:515–520.

Ishimaru, T., Hirabayashi, H., Kuwagata, T., Ogawa, T., and Kondo, M. (2012). The early-morning flowering trait of rice reduces spikelet sterility under windy and elevated temperature conditions at anthesis. Plant Prod. Sci. **15**:19–22.

Iwahori, S. (1965). High temperature injuries in tomato. IV. Engei Gakkai Zasshi **34**:33–41.

Jagadish, S.V.K., Craufurd, P.Q., and Wheeler, T.R. (2007). High temperature stress and spikelet fertility in rice (*Oryza sativa* L.). J. Exp. Bot. **58**:1627–1635.

Jagadish, S.V.K., Murty, M.V.R., and Quick, W.P. (2015). Rice responses to rising temperatures–challenges, perspectives and future directions. Plant Cell Environ. **38**:1686–1698.

Jagadish, S.V.K., Muthurajan, R., Oane, R., Wheeler, T.R., Heuer, S., Bennett, J., and Craufurd, P.Q. (2010). Physiological and proteomic approaches to address heat tolerance during anthesis in rice (*Oryza sativa* L.). J. Exp. Bot. **61**:143–156.

Jagadish, S.V.K. (2020). Heat stress during flowering in cereals - effects and adaptation strategies. New Phytol. **226**:1567–1572.

Jäger, K., Fábián, A., and Barnabás, B. (2008). Effect of water deficit and elevated temperature on pollen development of drought sensitive and tolerant winter wheat (*Triticum aestivum* L.) genotypes. Acta Biol. Szeged. **52**:67–71.

Jiang, Y., Lahlali, R., Karunakaran, C., Warkentin, T.D., Davis, A.R., and Bueckert, R.A. (2019). Pollen, ovules, and pollination in pea: success, failure, and resilience in heat. Plant Cell Environ. **42**:354–372.

Joshi, M.A., Faridullah, S., and Kumar, A. (2016). Effect of heat stress on crop phenology, yield and seed quality attributes of wheat (*Triticum aestivum* L.). J. Agrometeorol. **18**:206–215.

Julia, C., and Dingkuhn, M. (2013). Predicting temperature induced sterility of rice spikelets requires simulation of crop-generated microclimate. Eur. J. Agron. **49**:50–60.

Kakani, V.G., Reddy, K.R., Koti, S., Wallace, T.P., Prasad, P.V.V., Reddy, V.R., and Zhao, D. (2005). Differences in in vitro pollen germination and pollen tube growth of cotton cultivars in response to high temperature. Ann. Bot. **96**:59–67.

Karni, L., and Aloni, B. (2002). Fructokinase and hexokinase from pollen grains of bell pepper (*Capsicum annuum* L.): possible role in pollen

germination under conditions of high temperature and CO2 enrichment. Ann. Bot. **90**:607–612.

Keijzer, C., Leferink-ten Klooster, H., and Reinders, M. (1996). The mechanics of the grass flower: anther dehiscence and pollen shedding in maize. Ann. Bot. **78**:15–21.

Khabba, S., Ledent, J.-F., and Lahrouni, A. (2001). Maize ear temperature. Eur. J. Agron. **14**:197–208.

Khabba, S., Ledent, J.-F., Lahrouni, A., and meteorology, f. (1999). Development and validation of model of heat diffusion in maize ear. Agric. For. Meteorol. **97**:113–127.

Khan, A.H., Ma, Y., Wu, Y., Akbar, A., Shaban, M., Ullah, A., Deng, J., Khan, A.S., Chi, H., Zhu, L., et al. (2022a). High-temperature stress suppresses allene oxide cyclase 2 and causes male sterility in cotton by disrupting jasmonic acid signaling. The Crop Journal **11**:33–45.

Khan, A.H., Min, L., Ma, Y., Wu, Y., Ding, Y., Li, Y., Xie, S., Ullah, A., Shaban, M., Manghwar, H., et al. (2020). High day and night temperatures distinctively disrupt fatty acid and jasmonic acid metabolism, inducing male sterility in cotton. J. Exp. Bot. **71**:6128–6141.

Khan, A.H., Min, L., Ma, Y., Zeeshan, M., Jin, S., and Zhang, X. (2022b). High-temperature stress in crops: male sterility, yield loss, and potential remedy approaches. Plant Biotechnol. J. **21**:680–697.

Khan, S.U., Din, J.U., Qayyum, A., Jan, N.E., and Jenks, M.A. (2015). Heat tolerance indicators in Pakistani wheat (*Triticum aestivum* L.) genotypes. Acta Bot. Croat. **74**:109–121.

Khush, G.S., and Peng, S. (1998). Improving Yield Potential by Modifying Plant Type (No. 2169-2019-1604), Retrieved from. https://doi.org/10.22004/ag.econ.287587.

Kim, S.Y., Hong, C.B., and Lee, I. (2001). Heat shock stress causes stage-specific male sterility in Arabidopsis thaliana. J. Plant Res. **114**:301–307.

Koike, S., Yamaguchi, T., Ohmori, S., Hayashi, T., Yatou, O., and Yoshida, H. (2015). Cleistogamy decreases the effect of high temperature stress at flowering in rice. Plant Prod. Sci. **18**:111–117.

Kuriyama, H., and Fukuda, H. (2002). Developmental programmed cell death in plants. Curr. Opin. Plant Biol. **5**:568–573.

Laza, M.R.C., Sakai, H., Cheng, W., Tokida, T., Peng, S., and Hasegawa, T. (2015). Differential response of rice plants to high night temperatures imposed at varying developmental phases. Agric. For. Meteorol. **209-210**:69–77.

Lawas, L.M.F., Bheemanahalli, R., Solis, C.A., and Jagadish, S.V.K. (2018). Sheathed panicle phenotype (cv. Sathi) maintains normal spikelet fertility and grain filling under prolonged heat stress in rice. Crop Sci. **58**:1693–1705.

Li, S., Zhang, Y., Sun, Y., Zhang, G., Bai, J., Guo, J., Su, X., Du, H., Cao, X., Yang, J., et al. (2019). Identification and analysis of heat resistance of different germplasm resources of maize at flowering stage. Nutr. Diabetes **9**:28–37.

Lin, H., Chen, Y., Zhang, H., Fu, P., Fan, Z., and Watling, J. (2017). Stronger cooling effects of transpiration and leaf physical traits of plants from a hot dry habitat than from a hot wet habitat. Funct. Ecol. **31**:2202–2211.

Liu, L., Zou, Z., Qian, K., Xia, C., He, Y., Zeng, H., Zhou, X., Riemann, M., and Yin, C. (2017). Jasmonic acid deficiency leads to scattered floret opening time in cytoplasmic male sterile rice Zhenshan 97A. J. Exp. Bot. **68**:4613–4625.

Liu, M., Sheng, D., Liu, X., Wang, Y., Hou, X., Wang, Y., Wang, P., Guan, L., Dong, X., and Huang, S. (2022a). Dissecting heat tolerance and yield stability in maize from greenhouse and field experiments. J. Agron. Crop Sci. **208**:348–361.

Liu, M., Dong, X., Zhang, Y., Gu, M., Yu, Y., Xie, H., Yang, H., Yu, X., and Huang, S. (2022b). Heat stress on maize with contrasting genetic background: differences in flowering and yield formation. Agric. For. Meteorol. **319**:108934.

Liu, X., Gu, M., Lv, X., Sheng, D., Wang, X., Wang, P., and Huang, S. (2023). High temperature defense-related pathways, mediating lodicule expansion and spikelet opening in maize tassel. J. Exp. Bot. erad115.

Lohani, N., Singh, M.B., and Bhalla, P.L. (2020). High temperature susceptibility of sexual reproduction in crop plants. J. Exp. Bot. **71**:555–568.

Lord, E.M., and Russell, S.D. (2002). The mechanisms of pollination and fertilization in plants. Annu. Rev. Cell Dev. Biol. **18**:81–105.

Mackill, D., and Coffman, W. (1983). Inheritance of high temperature tolerance and pollen shedding in a rice cross. Zeitschrift fur Pflanzenzuchtung= Journal of plant breeding **91**:61–69. Retrieved from: https://agris.fao.org/agris-search/search.do?recordID=US201302165784.

Madan, P., Jagadish, S.V.K., Craufurd, P.Q., Fitzgerald, M., Lafarge, T., and Wheeler, T.R. (2012). Effect of elevated CO_2 and high temperature on seed-set and grain quality of rice. J. Exp. Bot. **63**:3843–3852.

Majumder, D., Kingra, P., and Kukal, S. (2016). Canopy temperature and heat use efficiency of spring maize under modified soil microenvironment. Ann. Agric. Res. **37**:225–235. Retrieved from: https://agris.fao.org/agris-search/search.do?recordID=IN2022000316.

Matsue, Y., Takasaki, K., and Abe, J. (2021). Water management for improvement of rice yield, appearance quality and palatability with high temperature during ripening period. Rice Sci. **28**:409–416.

Matsui, T., and Hasegawa, T. (2019). Effect of long anther dehiscence on seed set at high temperatures during flowering in rice (*Oryza sativa* L.). Sci. Rep. **9**:20363.

Matsui, T., and Omasa, K. (2002). Rice (*Oryza sativa* L.) cultivars tolerant to high temperature at flowering: anther characteristics. Ann. Bot. **89**:683–687.

Matsui, T., Omasa, K., and Horie, T. (2001). The difference in sterility due to high temperatures during the flowering period among japonica-rice varieties. Plant Prod. Sci. **4**:90–93.

Matsui, T., and Kagata, H. (2003). Characteristics of floral organs related to reliable self-pollination in rice (*Oryza sativa* L.). Ann. Bot. **91**:473–477.

Matsui, T., Kobayasi, K., Kagata, H., and Horie, T. (2005). Correlation between viability of pollination and length of basal dehiscence of the theca in rice undera hot-and-humid condition. Plant Prod. Sci. **8**:109–114.

Matsui, T., Kobayasi, K., Yoshimoto, M., Hasegawa, T., Tanaka, T.S., and Tian, X. (2021). Factors determining the occurrence of floret sterility in rice in a hot and low-wind paddy field in Jianghan Basin, China. Field Crop. Res. **267**:108161.

Matsui, T., Omasa, K., and Horie, T. (1997). High temperature-induced spikelet sterility of japonica rice at flowering in relation to air temperature, humidity and wind velocity conditions. Jpn. J. Crop Sci. **66**:449–455.

Matsui, T., Omasa, K., and Horie, T. (2000). High temperature at flowering inhibits swelling of pollen grains, a driving force for thecae dehiscence in rice (*Oryza sativa* L.). Plant Prod. Sci. **3**:430–434.

Miller, E.C. (1919). Development of the pistillate spikelet and fertilization in Zea mays. J. Agric. Res. **18**:255–267.

Mitchell, J., and Petolino, J. (1988). Heat stress effects on isolated reproductive organs of maize. J. Plant Physiol. **133**:625–628.

Mitchell, R.A.C., Mitchell, V.J., Driscoll, S.P., Franklin, J., and Lawlor, D.W. (1993). Effects of increased CO2 concentration and temperature on growth and yield of winter wheat at two levels of nitrogen application. Plant Cell Environ. **16**:521–529.

Mittler, R., Finka, A., and Goloubinoff, P. (2012). How do plants feel the heat? Trends Biochem. Sci. **37**:118–125.

Mohammed, A.R., and Tarpley, L. (2009). High nighttime temperatures affect rice productivity through altered pollen germination and spikelet fertility. Agric. For. Meteorol. **149**:999–1008.

Mohammed, A.R., and Tarpley, L. (2010). Effects of high night temperature and spikelet position on yield-related parameters of rice (Oryza sativa L.) plants. Eur. J. Agron. **33**:117–123.

Mohammed, A.R., and Tarpley, L. (2011). Effects of night temperature, spikelet position and salicylic acid on yield and yield-related parameters of rice (oryza sativa L.) plants. J. Agron. Crop Sci. **197**:40–49.

Mohammed, R., Cothren, J.T., and Tarpley, L. (2013). High night temperature and abscisic acid affect rice productivity through altered photosynthesis, respiration and spikelet fertility. Crop Sci. **53**:2603–2612.

Mol, R., Matthys-Rochon, E., and Dumas, C. (1994). The kinetics of cytological events during double fertilization in *Zea mays* L. Plant J. **5**:197–206.

Morice, C.P., Kennedy, J.J., Rayner, N.A., Winn, J.P., Hogan, E., Killick, R.E., Dunn, R.J.H., Osborn, T.J., Jones, P.D., and Simpson, I.R. (2021). An updated assessment of near-surface temperature change from 1850: the HadCRUT5 data set. Geophys. Res. Atmos. **126**.

Mu, X., Ma, Z., Zhang, L., Fu, J., Liu, T., Ding, Y., Xia, L., Zhang, F., Zhang, J., Qi, J., et al. (2022). Leaf photosynthetic fluorescence characteristics, pollination set and yield components of different tolerant/sensitive maize varieties in response to high flowering temperatures. Chin. J. Eco-Agric. **30**:57–71.

Muhlemann, J.K., Younts, T.L.B., and Muday, G.K. (2018). Flavonols control pollen tube growth and integrity by regulating ROS homeostasis during high-temperature stress. Proc. Natl. Acad. Sci. USA **115**:E11188–E11197.

Narayanan, S., Prasad, P.V.V., Fritz, A.K., Boyle, D.L., and Gill, B.S. (2015). Impact of high night-time and high daytime temperature stress on winter wheat. J. Agron. Crop Sci. **201**:206–218.

Nielsen, R. (2016). Silk Development and Emergence in Corn. Retrieved from. http://www.kingcorn.org/news/timeless/Silks.html.

Okada, T., Jayasinghe, J.E.A.R.M., Nansamba, M., Baes, M., Warner, P., Kouidri, A., Correia, D., Nguyen, V., Whitford, R., and Baumann, U. (2018). Unfertilized ovary pushes wheat flower open for cross-pollination. J. Exp. Bot. **69**:399–412.

Okuda, S., Tsutsui, H., Shiina, K., Sprunck, S., Takeuchi, H., Yui, R., Kasahara, R.D., Hamamura, Y., Mizukami, A., Susaki, D., et al. (2009). Defensin-like polypeptide LUREs are pollen tube attractants secreted from synergid cells. Nature **458**:357–361.

Oury, V., Tardieu, F., and Turc, O. (2016). Ovary apical abortion under water deficit is caused by changes in sequential development of ovaries and in silk growth rate in maize. Plant Physiol. **171**:986–996.

Pangga, I.B., Hanan, J., and Chakraborty, S. (2013). Climate change impacts on plant canopy architecture: implications for pest and pathogen management. Eur. J. Plant Pathol. **135**:595–610.

Parish, R.W., Phan, H.A., Iacuone, S., and Li, S.F. (2012). Tapetal development and abiotic stress: a centre of vulnerability. Funct. Plant Biol. **39**:553–559.

Peng, S., Huang, J., Sheehy, J.E., Laza, R.C., Visperas, R.M., Zhong, X., Centeno, G.S., Khush, G.S., and Cassman, K.G. (2004). Rice yields decline with higher night temperature from global warming. Proc. Natl. Acad. Sci. USA **101**:9971–9975.

Polowick, P.L., and Sawhney, V.K. (1988). High temperature induced male and female sterility in canola (*Brassica napus* L.). Ann. Bot. **62**:83–86.

Porter, J.R., and Gawith, M. (1999). Temperatures and the growth and development of wheat: a review. Eur. J. Agron. **10**:23–36.

Prasad, P.V.V., Pisipati, S.R., Mutava, R.N., and Tuinstra, M.R. (2008a). Sensitivity of grain sorghum to high temperature stress during reproductive development. Crop Sci. **48**:1911–1917.

Prasad, P.V.V., Craufurd, P.Q., Kakani, V.G., Wheeler, T.R., and Boote, K.J. (2001). Influence of high temperature during pre-and post-anthesis stages of floral development on fruit-set and pollen germination in peanut. Funct. Plant Biol. **28**:233–240.

Prasad, P.V.V., Bheemanahalli, R., and Jagadish, S.K. (2017). Field crops and the fear of heat stress-Opportunities, challenges and future directions. Field Crop. Res. **200**:114–121.

Prasad, P.V.V., and Djanaguiraman, M. (2014). Response of floret fertility and individual grain weight of wheat to high temperature stress: sensitive stages and thresholds for temperature and duration. Funct. Plant Biol. **41**:1261–1269.

Prasad, P.V.V., Pisipati, S.R., Ristic, Z., Bukovnik, U., and Fritz, A.K. (2008b). Impact of nighttime temperature on physiology and growth of spring wheat. Crop Sci. **48**:2372–2380.

Prasad, P.V.V., Pisipati, S.R., Momčilović, I., and Ristic, Z. (2011). Independent and combined effects of high temperature and drought stress during grain filling on plant yield and chloroplast EF-tu expression in spring wheat. J. Agron. Crop Sci. **197**:430–441.

Qin, Y., Yang, J., and Zhao, J. (2005). Calcium changes and the response to methyl jasmonate in rice lodicules during anthesis. Protoplasma **225**:103–112.

Sathishraj, R., Bheemanahalli, R., Ramachandran, M., Dingkuhn, M., Muthurajan, R., and Krishna, J.S.V. (2016). Capturing heat stress induced variability in spikelet sterility using panicle, leaf and air temperature under field conditions. Field Crop. Res. **190**:10–17.

Vj, R., and Inamdar, M.N. (2022). Impact of autonomous drone pollination in date palms. ijirss. **5**:297–305.

Rezaul, I.M., Baohua, F., Tingting, C., Weimeng, F., Caixia, Z., Longxing, T., and Guanfu, F. (2019). Abscisic acid prevents pollen abortion under high-temperature stress by mediating sugar metabolism in rice spikelets. Physiol. Plantarum **165**:644–663.

Riechmann, J.L., and Wellmer, F. (2014). Flower Development: Methods and Protocols (Springer).

Rieu, I., Wolters-Arts, M., Derksen, J., Mariani, C., and Weterings, K. (2003). Ethylene regulates the timing of anther dehiscence in tobacco. Planta **217**:131–137.

Rutley, N., Harper, J.F., and Miller, G. (2021). Reproductive resilience: putting pollen grains in two baskets. Trends Plant Sci. **27**:237–246.

Sadok, W., and Jagadish, S.V.K. (2020). The hidden costs of nighttime warming on yields. Trends Plant Sci. **25**:644–651.

Saini, H.S., and Aspinall, D. (1982). Abnormal sporogenesis in wheat (*Triticum aestivum* L.) induced by short periods of high temperature. Ann. Bot. **49**:835–846.

Saini, H.S., Sedgley, M., and Aspinall, D. (1983). Effect of heat stress during floral development on pollen tube growth and ovary anatomy in wheat (Triticum aestivum L.). Funct. Plant Biol. **10**:137–144.

Sakai, H., Cheng, W., Chen, C.P., and Hasegawa, T. (2022). Short-term high nighttime temperatures pose an emerging risk to rice grain failure. Agric. For. Meteorol. **314**:108779.

Sakata, T., and Higashitani, A. (2008). Male sterility accompanied with abnormal anther development in plants–genes and environmental stresses with special reference to high temperature injury. Int. J. Plant

Dev. Biol. 2. Retrieved from: http://www.globalsciencebooks.info/Online/GSBOnline/images/0806/IJPDB_2(1)/IJPDB_2(1)42-51o.pdf.

Salvador, D.W., and Pearce, R.B. (1988). Husk removal and its effects on maize grain yield. Crop Sci. **28**:961–964.

Sánchez, B., Rasmussen, A., and Porter, J.R. (2014). Temperatures and the growth and development of maize and rice: a review. Global Change Biol. **20**:408–417.

Sanders, P.M., Bui, A.Q., Le, B.H., and Goldberg, R.B. (2005). Differentiation and degeneration of cells that play a major role in tobacco anther dehiscence. Sex. Plant Reprod. **17**:219–241.

Santiago, J.P., and Sharkey, T.D. (2019). Pollen development at high temperature and role of carbon and nitrogen metabolites. Plant Cell Environ. **42**:2759–2775.

Scott, R.J., Spielman, M., and Dickinson, H.G. (2004). Stamen structure and function. Plant Cell **16**:S46–S60.

Semenov, M.A., and Shewry, P.R. (2011). Modelling predicts that heat stress, not drought, will increase vulnerability of wheat in Europe. Sci. Rep. **1**:66.

Sheng, D., Wang, Y., Huang, S., Tao, H., and Wang, P. (2020). Effect of high temperature on maize plant morphology and function, yield composition and grain nutrients. Journal of Maize Science **28**:86–92.

Shi, P., Zhu, Y., Tang, L., Chen, J., Sun, T., Cao, W., and Tian, Y. (2016). Differential effects of temperature and duration of heat stress during anthesis and grain filling stages in rice. Environ. Exp. Bot. **132**:28–41.

Shi, W., Yin, X., Struik, P.C., Solis, C., Xie, F., Schmidt, R.C., Huang, M., Zou, Y., Ye, C., and Jagadish, S.V.K. (2017). High day- and night-time temperatures affect grain growth dynamics in contrasting rice genotypes. J. Exp. Bot. **68**:5233–5245.

Shi, W., Li, X., Schmidt, R.C., Struik, P.C., Yin, X., and Jagadish, S.V.K. (2018). Pollen germination and in vivo fertilization in response to high-temperature during flowering in hybrid and inbred rice. Plant Cell Environ. **41**:1287–1297.

Shi, W., Muthurajan, R., Rahman, H., Selvam, J., Peng, S., Zou, Y., and Jagadish, K.S.V. (2013). Source-sink dynamics and proteomic reprogramming under elevated night temperature and their impact on rice yield and grain quality. New Phytol. **197**:825–837.

Shiferaw, B., Prasanna, B.M., Hellin, J., and Bänziger, M. (2011). Crops that feed the world 6. Past successes and future challenges to the role played by maize in global food security. Food Secur. **3**:307–327.

Siebert, S., Ewert, F., Eyshi Rezaei, E., Kage, H., and Graß, R. (2014). Impact of heat stress on crop yield—on the importance of considering canopy temperature. Environ. Res. Lett. **9**:044012.

Siebert, S., Webber, H., Zhao, G., and Ewert, F. (2017). Heat stress is overestimated in climate impact studies for irrigated agriculture. Environ. Res. Lett. **12**:054023.

Šim Škov, M.R., Daneva, A., Doll, N., Schilling, N., Cubría-Radío, M., Zhou, L., De Winter, F., Aesaert, S., De Rycke, R., Pauwels, L., et al. (2022). KIL1 terminates fertility in maize by controlling silk senescence. Plant Cell **34**:2852–2870.

Smith, L.M. (2019). The heat is on: maize pollen development after a heat wave. Plant Physiol. **181**:387–388.

Snider, J.L., Oosterhuis, D.M., and Kawakami, E.M. (2011a). Diurnal pollen tube growth rate is slowed by high temperature in field-grown Gossypium hirsutum pistils. J. Plant Physiol. **168**:441–448.

Snider, J.L., Oosterhuis, D.M., Loka, D.A., and Kawakami, E.M. (2011b). High temperature limits in vivo pollen tube growth rates by altering diurnal carbohydrate balance in field-grown Gossypium hirsutum pistils. J. Plant Physiol. **168**:1168–1175.

Snider, J.L., Oosterhuis, D.M., Skulman, B.W., and Kawakami, E.M. (2009). Heat stress-induced limitations to reproductive success in Gossypium hirsutum. Physiol. Plantarum **137**:125–138.

Solomon, S., Qin, D., Manning, M., Averyt, K., and Marquis, M. (2007). Climate change 2007-the physical science basis. In Working Group I Contribution to the Fourth Assessment Report of the IPCC (Cambridge university press).

Steinmeyer, F.T., Lukac, M., Reynolds, M.P., and Jones, H.E. (2013). Quantifying the relationship between temperature regulation in the ear and floret development stage in wheat (*Triticum aestivum* L.) under heat and drought stress. Funct. Plant Biol. **40**:700–707.

Stocker, T.F., Qin, D., Plattner, G.-K., Tignor, M.M., Allen, S.K., Boschung, J., Nauels, A., Xia, Y., Bex, V., and Midgley, P.M. (2014). Climate Change 2013: The Physical Science Basis. Contribution of Working Group I to the Fifth Assessment Report of IPCC the Intergovernmental Panel on Climate Change.

Stockle, C., and Dugas, W. (1992). Evaluating canopy temperature-based indices for irrigation scheduling. Irrigat. Sci. **13**:31–37. https://doi.org/10.1007/BF00190242.

Stone, P., and Nicolas, M. (1995). Effect of timing of heat stress during grain filling on two wheat varieties differing in heat tolerance. I. Grain growth. Funct. Plant Biol. **22**:927–934.

Stratonovitch, P., and Semenov, M.A. (2015). Heat tolerance around flowering in wheat identified as a key trait for increased yield potential in Europe under climate change. J. Exp. Bot. **66**:3599–3609.

Stuerz, S., Sow, A., Muller, B., Manneh, B., and Asch, F. (2014). Canopy microclimate and gas-exchange in response to irrigation system in lowland rice in the Sahel. Field Crop. Res. **163**:64–73.

Tadege, M., and Kuhlemeier, C. (1997). Aerobic fermentation during tobacco pollen development. Plant Mol. Biol. **35**:343–354.

Tang, R.-S., Zheng, J.-C., Jin, Z.-Q., Zhang, D.-D., Huang, Y.-H., and Chen, L.-G. (2007). Possible correlation between high temperature-induced floret sterility and endogenous levels of IAA, GAs and ABA in rice (*Oryza sativa* L.). Plant Growth Regul. **54**:37–43.

Dresselhaus, T., and Franklin-Tong, N. (2013). Male-female crosstalk during pollen germination, tube growth and guidance, and double fertilization. Mol. Plant **6**:1018–1036.

Tivoli, B., Calonnec, A., Richard, B., Ney, B., and Andrivon, D. (2013). Current knowledge on plant/canopy architectural traits that reduce the expression and development of epidemics. Eur. J. Plant Pathol. **135**:471–478.

Tranel, D.M. (2007). Morphology and Plasticity of Maize (*Zea mays* L.) Male Inflorescence Development and Pollen Production (Iowa State University).

Turc, O., Bouteillé, M., Fuad-Hassan, A., Welcker, C., and Tardieu, F. (2016). The growth of vegetative and reproductive structures (leaves and silks) respond similarly to hydraulic cues in maize. New Phytol. **212**:377–388.

Ullah, A., Nadeem, F., Nawaz, A., Siddique, K.H.M., Farooq, M., Subhan Qureshi, M., Khan, N.A., Ayari-Akkari, A., and Ahmed, D.A.E.M. (2022). Heat stress effects on the reproductive physiology and yield of wheat. J. Agron. Crop Sci. **208**:1–17.

Wang, M., Zhu, X., Peng, G., Liu, M., Zhang, S., Chen, M., Liao, S., Wei, X., Xu, P., Tan, X., et al. (2022). Methylesterification of cell-wall pectin controls the diurnal flower-opening times in rice. Mol. Plant **15**:956–972.

Wang, X., Zhu, B., Jiang, Z., and Wang, S. (2019a). Calcium-mediation of jasmonate biosynthesis and signaling in plants. Plant Sci. **287**:110192.

Wang, Y., Impa, S.M., Sunkar, R., and Jagadish, S.V.K. (2021a). The neglected other half - role of the pistil in plant heat stress responses. Plant Cell Environ. **44**:2200–2210.

Wang, Y., Sheng, D., Zhang, P., Dong, X., Yan, Y., Hou, X., Wang, P., and Huang, S. (2020a). High temperature sensitivity of kernel

formation in different short periods around silking in maize. Environ. Exp. Bot. **183**:104343.

Wang, Y., Tao, H., Tian, B., Sheng, D., Xu, C., Zhou, H., Huang, S., and Wang, P. (2019b). Flowering dynamics, pollen, and pistil contribution to grain yield in response to high temperature during maize flowering. Environ. Exp. Bot. **158**:80–88.

Wang, Y., Tao, H., Zhang, P., Hou, X., Sheng, D., Tian, B., Wang, P., and Huang, S. (2020b). Reduction in seed set upon exposure to high night temperature during flowering in maize. Physiol. Plantarum **169**:73–82.

Wang, Y., Wang, L., Zhou, J., Hu, S., Chen, H., Xiang, J., Zhang, Y., Zeng, Y., Shi, Q., Zhu, D., et al. (2019). Research progress on heat stress of rice at flowering stage. Rice Sci. **26**:1–10.

Wang, Y., Liu, X., Hou, X., Sheng, D., Dong, X., Gao, Y., Wang, P., and Huang, S. (2021b). Maximum lethal temperature for flowering and seed set in maize with contrasting male and female flower sensitivities. J. Agron. Crop Sci. **207**:679–689.

Wang, Z., Gu, Y., and Gao, Y. (1989). Studies on the mechanism of rice glume-opening: II. Effect of CO2 on glume-opening. Acta Agron. Sin. **15**:59–66. Retrieved from: https://agris.fao.org/agris-search/search.do?recordID=CN9082360.

Wang, Z., Gu, Y., and Gao, Y. (1991). Studies on the mechanism of the anthesis of rice-Structure of the lodicule and changes of its contents during flowering. Acta Agron. Sin. **17**:96–101. Retrieved from: https://agris.fao.org/agris-search/search.do?recordID=CN9280694.

Wang, Y., Sheng, D., Hou, X., Zhang, P., Liu, X., Wang, P., and Huang, S. (2023). Positive response of maize husk traits for improving heat tolerance during flowering by alleviating husk inside temperature. Agric. For. Meteorol. **335**:109455.

Wassmann, R., Jagadish, S.V.K., Heuer, S., Ismail, A., Redona, E., Serraj, R., Singh, R.K., Howell, G., Pathak, H., and Sumfleth, K. (2009). Climate change affecting rice production: the physiological and agronomic basis for possible adaptation strategies. Adv. Agron. **101**:59–122.

Webber, H., Ewert, F., Kimball, B., Siebert, S., White, J.W., Wall, G., Ottman, M.J., Trawally, D., and Gaiser, T. (2016). Simulating canopy temperature for modelling heat stress in cereals. Environ. Model. Software **77**:143–155.

Wędzony, M., and Van Lammeren, A.A.M. (1996). Pollen tube growth and early embryogenesis in wheat× maize crosses influenced by 2, 4-D. Ann. Bot. **77**:639–647.

Weterings, K., and Russell, S.D. (2004). Experimental analysis of the fertilization process. Plant Cell **16**:S107–S118.

Weng, X., Xu, J., Zhao, J., Huang, Y., Bian, X., and Wang, J. (2022). Study on seed production and pollination of Indica-japonica hybrid rice assisted by multi-rotor UAV. Journal of Chinese Agricultural Mechanization **43**:24–29.

Wheeler, T.R., Batts, G.R., Ellis, R.H., Hadley, P., and Morison, J.I.L. (1996a). Growth and yield of winter wheat (*Triticum aestivum*) crops in response to CO_2 and temperature. J. Agric. Sci. **127**:37–48.

Wheeler, T., Hong, T., Ellis, R., Batts, G., Morison, J., and Hadley, P. (1996b). The duration and rate of grain growth, and harvest index, of wheat (*Triticum aestivum* L.) in response to temperature and CO_2. J. Exp. Bot. **47**:623–630.

Wilson, Z.A., Song, J., Taylor, B., and Yang, C. (2011). The final split: the regulation of anther dehiscence. J. Exp. Bot. **62**:1633–1649.

Wu, C., Cui, K., Wang, W., Li, Q., Fahad, S., Hu, Q., Huang, J., Nie, L., and Peng, S. (2016). Heat-induced phytohormone changes are associated with disrupted early reproductive development and reduced yield in rice. Sci. Rep. **6**:34978.

Wu, C.S., Chen, Y.H., Chen, C.L., Chien, S.K., Syifa, N., Hung, Y.C., Cheng, K.J., Hu, S.C., Lo, P.T., Lin, S.Y., et al. (2019). Enclosed stigma contributes to higher spikelet fertility for rice (*Oryza sativa* L.) subjected to heat stress. The Crop Journal **19**:335–349.

Wu, C., Cui, K., Tang, S., Li, G., Wang, S., Fahad, S., Nie, L., Huang, J., Peng, S., and Ding, Y. (2020). Intensified pollination and fertilization ameliorate heat injury in rice (*Oryza sativa* L.) during the flowering stage. Field Crops Res. **252**:107795.

Wu, S., and Tsai, C. (1965). Cytological studies of the double fertilization in rice. J. Integr. Plant Biol. **13**. Retrieved from. https://www.jipb.net/EN/abstract/abstract24913.shtml.

Xiao, Y., Chen, Y., Charnikhova, T., Mulder, P.P.J., Heijmans, J., Hoogenboom, A., Agalou, A., Michel, C., Morel, J.B., Dreni, L., et al. (2014). OsJAR1 is required for JA-regulated floret opening and anther dehiscence in rice. Plant Mol. Biol. **86**:19–33.

Xu, F.-Q., Li, X.-R., and Ruan, Y.-L. (2008). RNAi-mediated suppression of hexokinase gene OsHXK10 in rice leads to non-dehiscent anther and reduction of pollen germination. Plant Sci. **175**:674–684.

Xu, J., Henry, A., and Sreenivasulu, N. (2020). Rice yield formation under high day and night temperatures-A prerequisite to ensure future food security. Plant Cell Environ. **43**:1595–1608.

Yadav, S.K., Sinha, D.K., Ahmed, A., Azhar, T., Sinha, M., Vanaja, M., and Maheswari, M. (2015). Genotypic variation in physiological traits under high temperature stress in maize. Indian J. Surg. Oncol. **6**:119–122.

Yan, C., Ding, Y., Wang, Q., Liu, Z., Li, G., Muhammad, I., and Wang, S. (2010). The impact of relative humidity, genotypes and fertilizer application rates on panicle, leaf temperature, fertility and seed setting of rice. J. Agric. Sci. **148**:329–339.

Yan, H., Zhang, B., Zhang, Y., Chen, X., Xiong, H., Matsui, T., and Tian, X. (2017). High temperature induced glume closure resulted in lower fertility in hybrid rice seed production. Front. Plant Sci. **7**:1960.

Yang, J., Fei, K., Chen, J., Wang, Z., Zhang, W., and Zhang, J. (2020). Jasmonates alleviate spikelet-opening impairment caused by high temperature stress during anthesis of photo-thermo-sensitive genic male sterile rice lines. Food Energy Secur. **9**:15.

Yasuor, H., Abu-Abied, M., Belausov, E., Madmony, A., Sadot, E., Riov, J., and Rubin, B. (2006). Glyphosate-induced anther indehiscence in cotton is partially temperature dependent and involves cytoskeleton and secondary wall modifications and auxin accumulation. Plant Physiol. **141**:1306–1315.

Yoshida, H. (2012). Is the lodicule a petal: molecular evidence? Plant Sci. **184**:121–128.

Yoshida, S., Satake, T., and Mackill, D. (1981). High-temperature Stress in Rice [study Conducted at IRRI, Philippines] (IRRI Research Paper Series).

Yu, K., Liu, Y., Li, Y., Sun, N., Zhan, J., You, D., Niu, L., Li, C., and Liu, T. (2016). Screening and comprehensive evaluation of heat-tolerance of maize hybrids in flowering stage. Journal of Maize Science **24**:62–71.

You, R., and Jensen, W.A. (1985). Ultrastructural observations of the mature megagametophyte and the fertilization in wheat (*Triticum aestivum*). Can. J. Bot. **63**:163–178.

Zeng, X., Zhou, X., Zhang, W., Murofushi, N., Kitahara, T., and Kamuro, Y. (1999). Opening of rice floret in rapid response to methyl jasmonate. J. Plant Growth Regul. **18**:153–158.

Zhao, L., Zhao, C.-F., Zhou, L.-H., Lin, J., Zhao, Q.-Y., Zhu, Z., Chen, T., Yao, S., Hasegawa, T., Matsui, T., et al. (2016). QTL mapping of dehiscence length at the basal part of thecae related to heat tolerance of rice (*Oryza sativa* L.). Euphytica **209**:715–723.

Zhang, Y., Tang, Q., Peng, S., Zou, Y., Chen, S., Shi, W., Qin, J., and Laza, M.R.C. (2013). Effects of high night temperature on yield and

agronomic traits of irrigated rice under field chamber system condition. Aust. J. Crop. Sci. **7**:7–13.

Zhang, C., Li, G., Chen, T., Feng, B., Fu, W., Yan, J., Islam, M.R., Jin, Q., Tao, L., and Fu, G. (2018). Heat stress induces spikelet sterility in rice at anthesis through inhibition of pollen tube elongation interfering with auxin homeostasis in pollinated pistils. Rice **11**:14.

Zhang, Y.-C., He, R.-R., Lian, J.-P., Zhou, Y.-F., Zhang, F., Li, Q.-F., Yu, Y., Feng, Y.-Z., Yang, Y.-W., Lei, M.-Q., et al. (2020). OsmiR528 regulates rice-pollen intine formation by targeting an uclacyanin to influence flavonoid metabolism. Proc. Natl. Acad. Sci. USA **117**:727–732.

Zhang, Z., Hu, M., Xu, W., Wang, Y., Huang, K., Zhang, C., and Wen, J. (2021). Understanding the molecular mechanism of anther development under abiotic stresses. Plant Mol. Biol. **105**:1–10.

Zhao, C., Liu, B., Piao, S., Wang, X., Lobell, D.B., Huang, Y., Huang, M., Yao, Y., Bassu, S., Ciais, P., et al. (2017). Temperature increase reduces global yields of major crops in four independent estimates. Proc. Natl. Acad. Sci. USA **114**:9326–9331.

Zhou, L.-Z., and Dresselhaus, T. (2022). Multiple roles of ROS in flowering plant reproduction. In Advances in Botanical Research (Academic Press).

Zhu, T., De Lima, C.F.F., and De Smet, I. (2021). The heat is on: how crop growth, development and yield respond to high temperature. J. Exp. Bot. **72**: erab308–7373.

Zhu, X., Chen, J., Huang, S., Li, W., Penuelas, J., Chen, J., Zhou, F., Zhang, W., Li, G., Liu, Z., et al. (2022). Manure amendment can reduce rice yield loss under extreme temperatures. Commun. Earth Environ. **3**:147.

Zweifel, R., Sterck, F., Braun, S., Buchmann, N., Eugster, W., Gessler, A., Häni, M., Peters, R.L., Walthert, L., Wilhelm, M., et al. (2021). Why trees grow at night. New Phytol. **231**:2174–2185.

Plant Communications, Volume *4*

Supplemental information

From the floret to the canopy: High temperature tolerance during flowering

Mayang Liu, Yuhan Zhou, Jiaxin Sun, Fen Mao, Qian Yao, Baole Li, Yuanyuan Wang, Yingbo Gao, Xin Dong, Shuhua Liao, Pu Wang, and Shoubing Huang

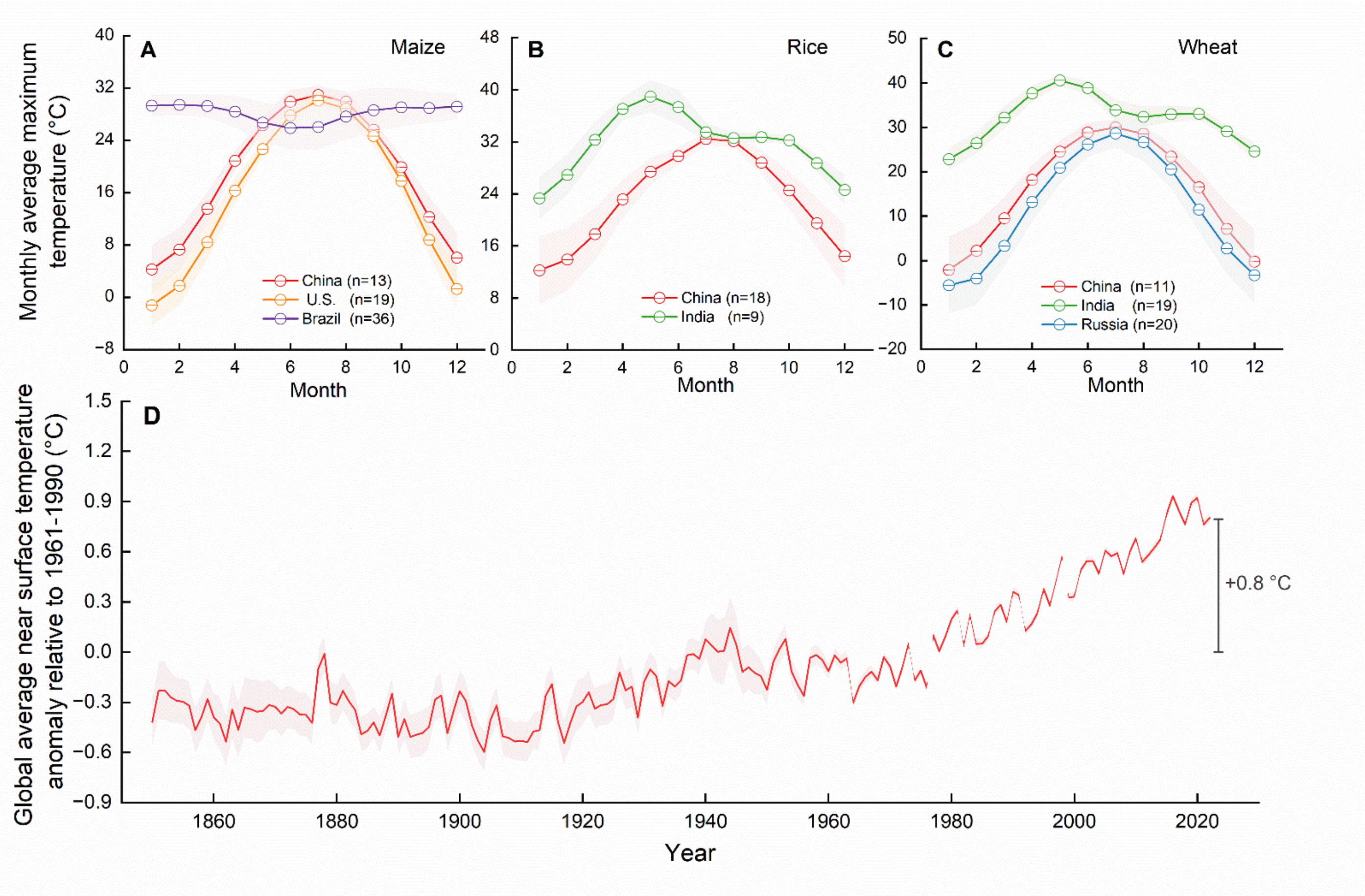

Supplementary Figure S1. Maximum temperature varies on monthly level and global average near surface temperature rises year-by-year. (A-C) Monthly average maximum temperature fluctuation in the major production countries of maize (China, U. S. and Brazil), rice (China and India) and wheat (China, India and Russia). Data were plotted from the mean of monthly recorded maximum temperature from 1960 to 2018 in local meteorological stations (the "n" represents the number of stations selected in each country). Data were retrieved from https://www.worldclim.org/data/monthlywth. The shaded areas indicated the margin of standard deviation (SD) in averaging the monthly mean maximum temperatures recorded at several stations in each country. (D) Global average near surface temperature anomaly relative to 1961-1990. The HadCRUT5 near surface temperature dataset provided by Met Office Hadley Centre and Climatic Research Unit (Morice et al., 2012), and retrieved from https://hadleyserver.metoffice.gov.uk/hadcrut5. Recorded interval of 1850-2022. The light pink area and lower limit and upper limit denote the 95% confidence interval of the HadCRUT5 dataset.

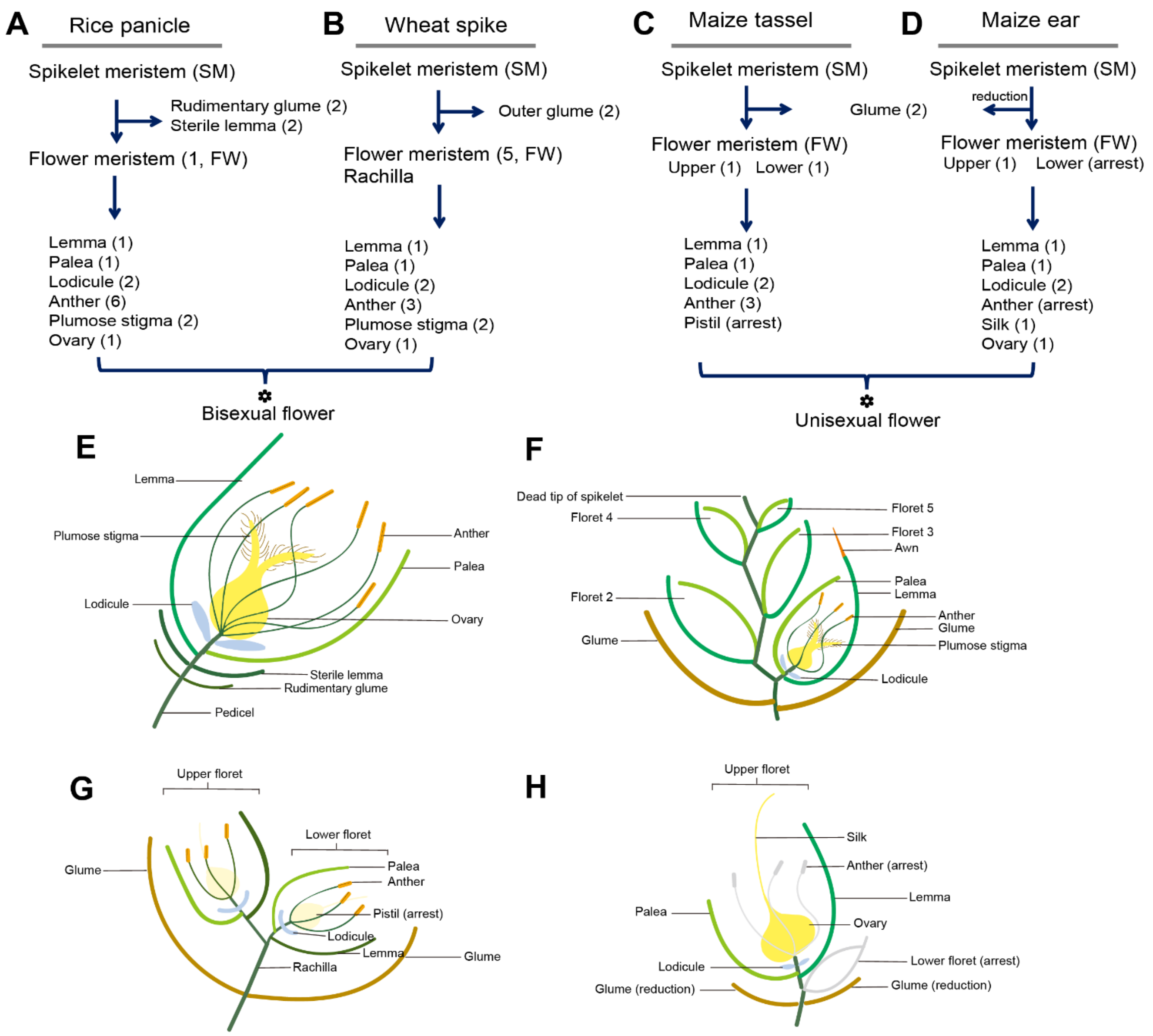

Supplementary Figure S2. Spikelet (or floret) development in rice, wheat and maize under normal growth environment. (A – D) Meristem transition and lateral organ differentiation of spikelet on the rice panicle, wheat spike, maize tassel and ear, which are summarized based on Riechmann & Wellmer (2014), with modifications. (E – H) Schematic representation of the bisexual floret in rice (E) and wheat (F), and the unisexual floret in maize tassel (G) and ear (H), which are drawn based on McSteen, Laudencia-Chingcuanco, & Colasanti (2000), Thompson & Hake (2009), Morojele & Burger (2009), with modifications. The florets are composed of the glume, lemma, palea, lodicules, and male and female reproduction organ (i.e., stamens and pistil). During sexual differentiation, some of the floret tissues undergo different degrees of degeneration or even abortion, e.g., rudimentary glume, and sterile lemma of another abortion floret in rice; arrested pistil of unisexual floret in maize tassel; reduced glume, arrested lower floret, and arrested anther and style of upper floret in maize ear.

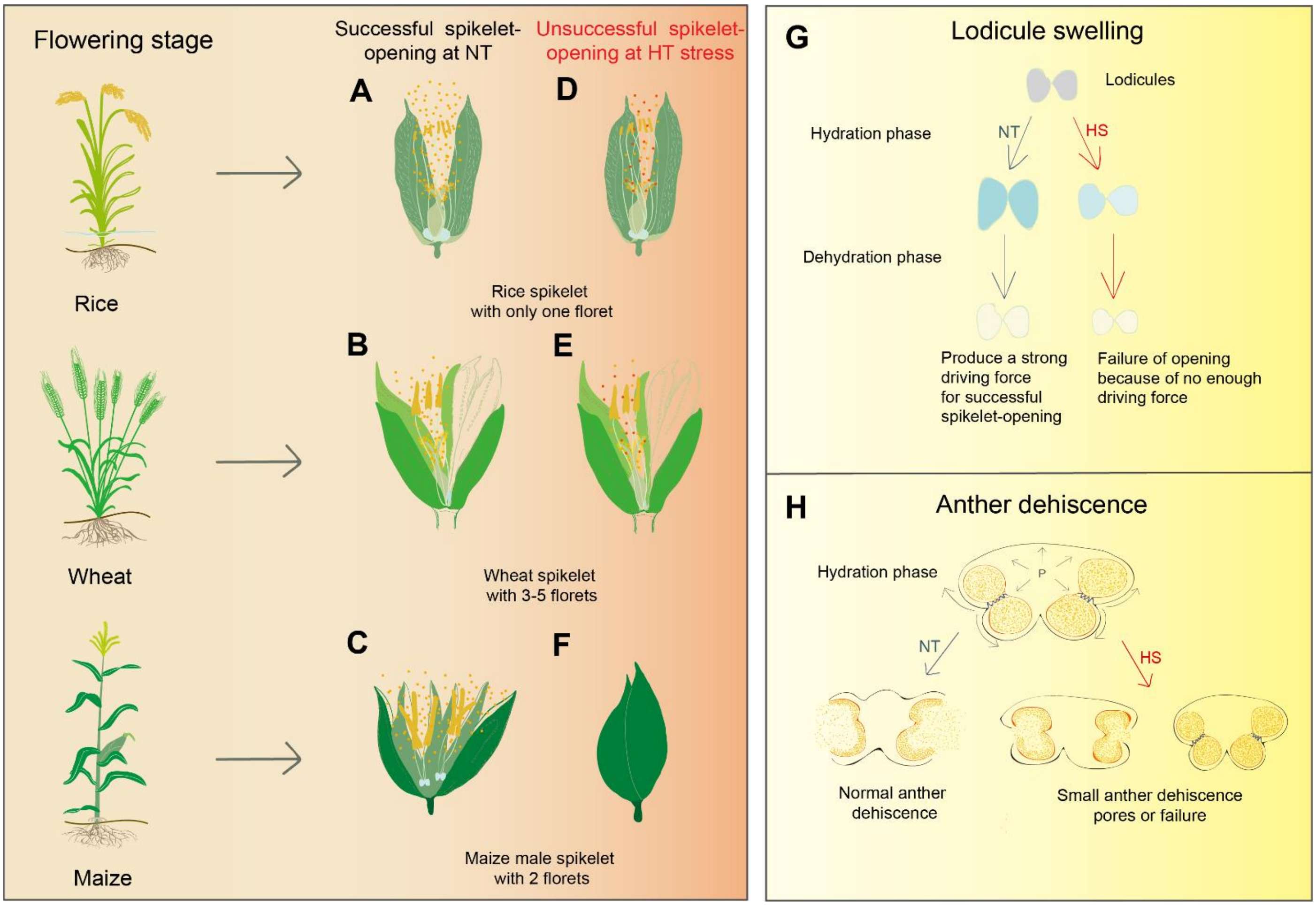

Supplementary Figure S3. Moderate or extreme high temperature (HT) stress affects spikelet-opening and pollen-shedding of rice, wheat and maize at flowering stage. (A-F) illustrate the morphological differences of the spikelet-opening at normal temperature (NT) and HT stress, respectively. HT stress results in unsuccessful spikelet-opening, including smaller opening angle or even opening failure, defective anther dehiscence, non-viable pollen grains (viable: pale yellow; nonviable: red) and reduced pollen shedding number. (G) depicts the distinct hydration and dehydration states of spikelet (floret) lodicules under NT and HT stress. During the spikelet-opening, the lodicules absorb enough water and swell resulting in a strong driving force to sperate lemma and palea of spikelet, which is significantly limited under HT stress. (H) clearly illustrates the pressure characteristic of anther at hydration stage (the driving force for dehiscence) and the various extents of anther dehiscence under NT and HT stress. HT stress induces the defective anther dehiscence with small dehiscence pore or even failure. This graphic was drawn based on Beauzamy, Nakayama, & Boudaoud (2014), with modifications.

Supplementary Table S1. Parameters of nonlinear Boltzmann model for fitting seed set ratio (%) of rice, wheat, and maize in response to high daytime and nighttime temperatures.

Parameter	SS_HDT			SS_HNT		
	Rice	Wheat	Maize	Rice	Wheat	Maize
SS_A_1	81.3(1.5)	82.4(1.7)	80.3(1.4)	81.9(1.1)	82.1(2.0)	84.6(1.2)
SS_A_2	32.0(2.9)	57.0(1.8)	37.9(3.6)	31.1(3.0)	62.6(5.2)	64.4(2.3)
$TT_{cri}\_X_0$	37.2(0.2)	27.3(0.5)	37.9(0.4)	31.2(0.2)	19.6(2.7)	27.3(1.6)
R^2	0.55	0.42	0.60	0.66	0.32	0.47
p_value	*<0.000*	*<0.000*	*<0.000*	*<0.000*	*<0.000*	*<0.000*
n	386	175	202	205	56	76

Note: Values in the bracket are the standard error. The critical temperature threshold is the temperature level during flowering that can significantly change the seed set ratio. Boltzmann model is as follows:

$$y = A_2 + (A_1 - A_2)/(1 + \exp((x - x_0)/dx)$$

where A_1 and A_2 are the initial and final values (i.e., SS ratio) of the fit curve, X is the dependent variable (i.e., temperature level), and X_0 is the threshold coefficient (i.e., TT_{cri}).

Supplementary Table S2. Seed set ratio (%) of rice, wheat, and maize when temperature exceeds high daytime and nighttime temperature thresholds (HDT and HNT) in different duration (i.e., hour, day, and week).

Species	Temperature threshold	Max. temperature	Duration of treatment	Seed set (%)	Number of data
**Rice**	HDT>37.2°C	37.5°C	6d	66.3% (44.1~85.3%)	9
		38°C	4-6h	53.5% (25.2~82.6%)	16
			2-6d	46.5% (3.8~85.7%)	48
		39°C	2-4d	15.9% (0.6~71.5%)	29
			2w	58.7% (31.5~84.0%)	12
		40°C	5-6d	35.8% (13.2~62.1%)	15
			1-2w	24.3% (10.0~40.2%)	24
	HNT>31.2°C	32°C	3d	66.6% (66.5~66.6%)	2
			>2w	42.7% (11.8-86.1%)	20
		35°C	>2w	31.5% (10.9-59.7%)	20
**Wheat**	HDT>27.3°C	30°C	5d	90.3% (86.4~99.0%)	5
		35°C	6-7d	77.9% (62.2~89.7%)	3
		31~35°C	>1w	58.5% (20.2~79.6%)	58
	HNT>19.6°C	20~24°C	<1w	72.4% (64.9~89.3%)	5
		20~23°C	>1w	65.0% (60.4~69.7%)	2
**Maize**	HDT>37.9°C	38~39°C	>2w	48.0% (7.6~81.5%)	62
		40~42°C	5d	67.0% (56.6~75.3%)	6
			>2w	29.3% (3.0~71.1%)	21
	HNT>27.3°C	30°C	2w	64.5% (58.2~74.0%)	10

Note: seed set ratio (%) is the mean value of multiple data across the above conditions. Values in bracket are the range of the mean

Supplementary Table S3. Flowering trait, grain yield, and fertility of spikelet and/or floret of rice, wheat and maize under different temperature levels around flowering. Data are derived from temperature-controlled experiments.

Species	Trait	Experimental conditions	High temperature region (Day/night temperature)	Stage of treatment	Duration of temperature	Grain yield (GY)/spikelet/floret fertility (SF/FF) loss relative to normal	Reference
Rice	Spikelet-opening	phytotron	39°C for 2h/26°C	*Ca.* 50% of plans at heading	48h	21%~53.4% of GY	Chen et al., 2020;
	Flowering Peak	Growth cabinet	35°C, 38°C for 11h	Anthesis	1d	7% per °C > 29.6°C or 2.4% per °C >33°C of SF	Jagadish et al., 2007
	Anther dehiscence	Sun-lit phytotron	37.5°C for 6h/26°C	Middle heading	6d	9.3%~49.3% of SF	Matsui et al., 2002
		Growth chamber	37°C, 39°C for 7h	After flowering	3d	/	Matsui et al., 2019
	Stigma elongation	Temperature-controlling greenhouse	35-36°C for 3h/24-27°C	Before anthesis	7d	16.1%~ 38.4% of SF at whole panicles	Wu et al.,2019;
	Pollen production	Temperature-gradient greenhouse	Ambient+5°C	Sowing	/	9.3%~77.7% of SF	Prasad et al., 2006
	Pollination	Sun-lit phytotron	40°C for 6h/26°C	Middle heading	6d	43.1%~79.7% of SF	Matsui et al., 2001

**Wheat**	Time of flowering	Growth chamber	34°C for 7h/16°C	Start of flowering	10d	22%~38% of GY	Aiqing et al., 2018
	Floret morphology	Growth chamber	36°C for 8h/26°C	Onset of booting	5d	FF decreased from 85% at 24°C to 0% at 35°C	Prasad et al., 2014
	Male morphology	Controlled environment cabinet	30°C/30°C, 30°C/20°C	Onset of meiosis in the pollen mother cells	3d	25% of grain set at 30°C/20°C; 37.4% at 30°C/30°C	Saini et al., 1982, 1984,
	Female morphology and fertility	Controlled environment cabinet	30°C/30°C	Onset of meiosis in the pollen mother cells	3d	/	Saini et al., 1983
**Maize**	Flowering dynamics	Environment-controlled greenhouse	40°C for 2h/30°C	V14 stage	14d	73.6% of GY	Wang et al., 2019
		Sun-lit phytotron	40°C at ear level for 4h	15 days before anthesis	~15d	51.8% of kernels loss	Edreira et al., 2011
	Pollen development	Growth chamber	35°C/25°C	Tetrad stage of pollen development	2d	A strong reduction in the number of seeds per cob	Begcy et al., 2019
	Pollen germination						
	Pollen-stigma interaction	Incubator	38°C/22°C	The application of pollen to the silks	2d	15%~18.6% of swelling kernels and 14.1%~17% of formed kernels	Mitchell et al., 1988

	Pollination	Environmental chamber	40°C	/	2 to 24 h after pollination	No fertilization occurs after 6 h of stress	Dupuis et al., 1990
